# TP-0903 is active in models of drug-resistant acute myeloid leukemia

**DOI:** 10.1172/jci.insight.140169

**Published:** 2020-12-03

**Authors:** Jae Yoon Jeon, Daelynn R. Buelow, Dominique A. Garrison, Mingshan Niu, Eric D. Eisenmann, Kevin M. Huang, Megan E. Zavorka Thomas, Robert H. Weber, Clifford J. Whatcott, Steve L. Warner, Shelley J. Orwick, Bridget Carmichael, Emily Stahl, Lindsey T. Brinton, Rosa Lapalombella, James S. Blachly, Erin Hertlein, John C. Byrd, Bhavana Bhatnagar, Sharyn D. Baker

**Affiliations:** 1 Division of Pharmaceutics and Pharmacology, College of Pharmacy,; 2 Sumitomo Dainippon Pharma Oncology,; 3 Division of Hematology, Department of Internal Medicine, and; 4 Comprehensive Cancer Center, The Ohio State University (OSU), Columbus, Ohio, USA.

**Keywords:** Hematology, Oncology, Drug therapy, Leukemias

## Abstract

Effective treatment for AML is challenging due to the presence of clonal heterogeneity and the evolution of polyclonal drug resistance. Here, we report that TP-0903 has potent activity against protein kinases related to STAT, AKT, and ERK signaling, as well as cell cycle regulators in biochemical and cellular assays. In vitro and in vivo, TP-0903 was active in multiple models of drug-resistant *FLT3* mutant AML, including those involving the F691L gatekeeper mutation and bone marrow microenvironment–mediated factors. Furthermore, TP-0903 demonstrated preclinical activity in AML models with *FLT3*-ITD and common co-occurring mutations in *IDH2* and *NRAS* genes. We also showed that TP-0903 had ex vivo activity in primary AML cells with recurrent mutations including *MLL*-PTD, *ASXL1*, *SRSF2*, and *WT1*, which are associated with poor prognosis or promote clinical resistance to AML-directed therapies. Our preclinical studies demonstrate that TP-0903 is a multikinase inhibitor with potent activity against multiple drug-resistant models of AML that will have an immediate clinical impact in a heterogeneous disease like AML.

## Abstract 2

TP-0903, a multikinase inhibitor, demonstrates preclinical activity in models of drug-resistant AML, including those involving FLT3 mutations, bone marrow microenvironment-mediated factors and recurrent mutations.

## Graphical abstract

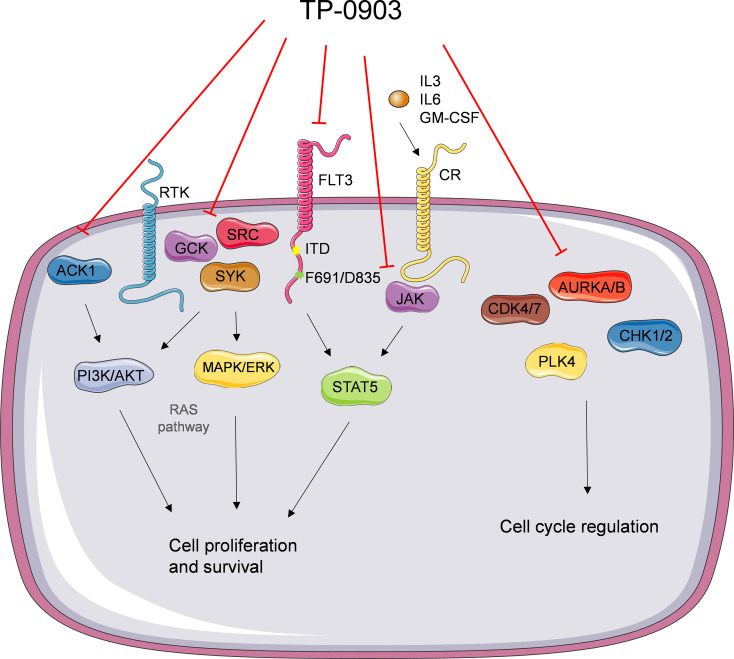

## Introduction

Acute myeloid leukemia (AML) is characterized by the aberrant proliferation and impaired differentiation of myeloid cells. Additionally, it is a heterogeneous disease with multiple molecular abnormalities. Recent studies have shown that recurrent gene mutations coexist in multiple leukemic clones within individual patients, with complex architecture and clonal evolution, all of which contribute to disease prognosis, as well as clinical drug resistance (1–3).

Recent developments in genomic sequencing technologies have made it possible to deep sequence AML samples and profile the mutational spectrum. With this information, guidelines have been developed to assign patients to prognostic risk groups based on their individual genetic alterations and provide guidance to tailor treatments to risk category (4). For example, *ASXL1* mutations commonly occur in older patients and associate with adverse outcomes (1, 4). *FLT3-*ITD–mutated AML is considered an aggressive disease with high risk of early disease relapse and poor survival (5–7). *NPM1* mutations are considered recurrent in AML, and while harboring *NPM1* mutations align with a favorable risk category, it is no longer considered favorable in the presence of an *FLT3*-ITD mutation (4). Although certain genetic mutations have not been classified in an unfavorable risk category, mutations in epigenetic modifiers (*DNMT3A*, *IDH1/2*, *EZH2*, *BCOR*), transcription factors (*WT1*, *MLL-*PTD), signaling genes (*KRAS*, *NRAS*), and splicing factors (*SRSF2*) among others have been associated with poorer risk, early relapse, or treatment resistance (4, 8–16). As such, clonal heterogeneity of the disease makes AML difficult to treat, and agents with selective activity are prone to clonal selection and drug resistance.

In preclinical studies, TP-0903 — a small molecule originally developed as an AXL inhibitor (17) — demonstrated anticancer activity in chronic lymphocytic leukemia (CLL), invasive breast cancer, and neuroblastoma (18–20). Currently, TP-0903 is undergoing evaluation in a first-in-human study in patients with advanced solid tumors (CinicalTrials.gov; NCT02729298) and in a phase 1/2 study in patients with previously treated CLL (NCT03572634). Here, we show that TP-0903 has potent activity against receptor tyrosine kinases and intracellular kinases related to STAT, AKT, and ERK signaling, as well as cell cycle regulators. We demonstrate the preclinical activity of TP-0903 in multiple models representing high-risk and drug-resistant subtypes of AML with a range of recurrent co-occurring mutations of prognostic relevance.

## Results

### TP-0903 is a multikinase inhibitor.

**Figure 1:**
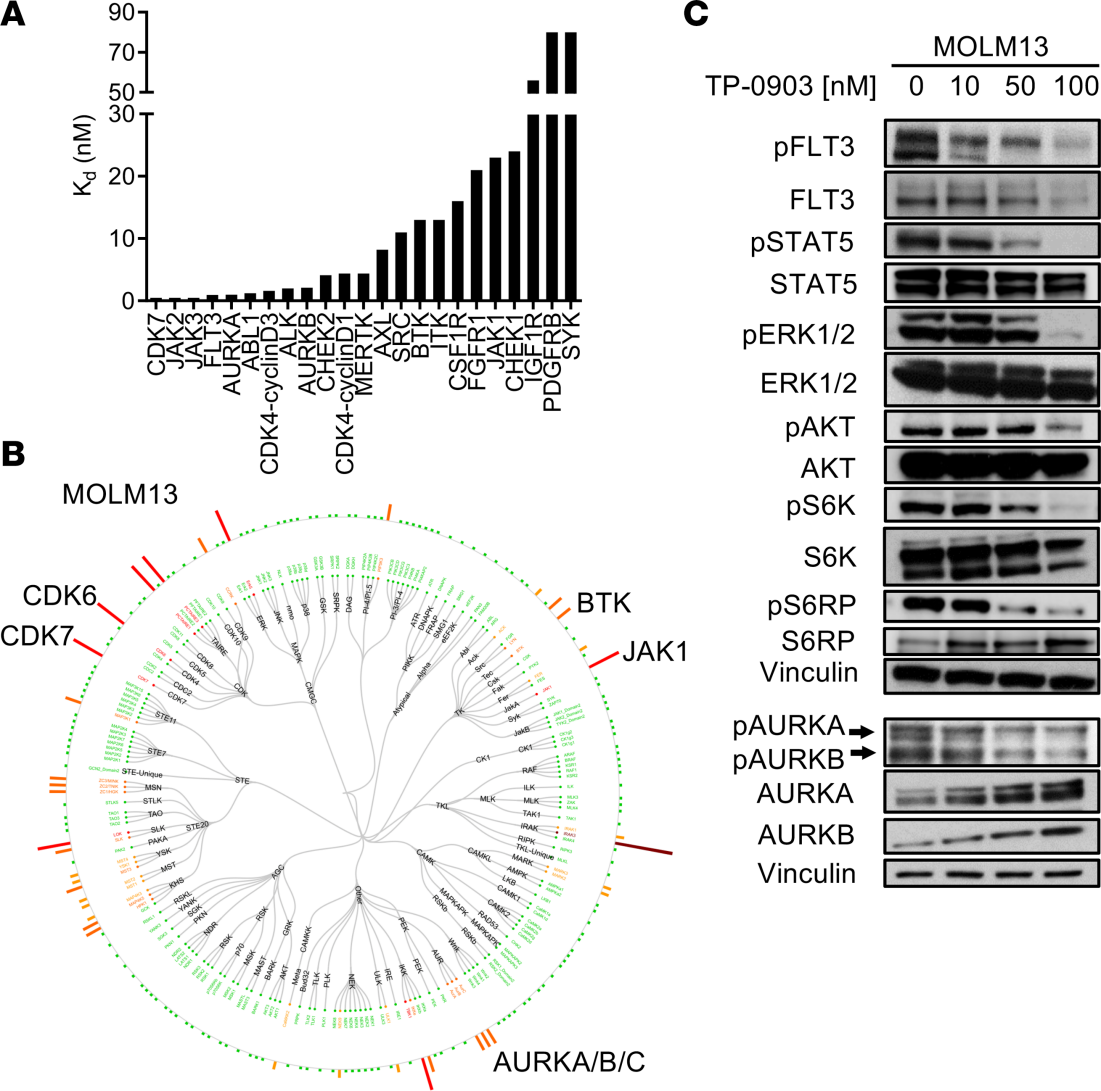
Kinase inhibition profile of TP-0903. (**A**) Inhibition of a panel of kinases by TP-0903 in a KdELECT assay. K_D_ (inhibitor binding constant) represented in nM. (**B**) Dendogram of native kinase inhibition in MOLM13 cells treated with 100 nM TP-0903 for 2 hours in a KiNativ assay. All kinases detected are shown and grouped based on classification. Inhibition is relative to the DMSO control indicated with a spectrum of dark red (>90%), red (75%–90%), orange (50–75%), yellow (35%–50%), and green (no change) bars. (**C**) Signaling inhibition in MOLM13 cells treated with DMSO or increasing concentrations of TP-0903 for 4 hours. Western blot analysis was performed on whole-cell lysates run on parallel gels with the indicated antibodies. A loading control was included with each gel, and a representative blot is shown; the top Vinculin blot served as a loading control for pERK1/2, and the bottom Vinculin blot served as a loading control for pAURKA/B. Data are representative of 2–3 independent experiments.

In a previous screen consisting of 40 kinases, 200 nM of TP-0903 inhibited 11 kinases by > 50% (17). We evaluated the inhibition of these and additional kinases by TP-0903 in a KdELECT binding assay with resultant K_D_ values that were < 5 nM for ABL1, ALK, AURKA, AURKB, CDK4, CDK7, CHEK2, FLT3, JAK2, JAK3, MERTK, PLK4, and TYK2. Other relevant kinase targets with K_D_ values < 100 nM were AXL (8.2 nM), BTK (13 nM), CHEK1 (24 nM), CSF1R (16 nM), FGFR1 (21 nM), and JAK1 (23 nM) (Figure 1A and Supplemental Figure 1A; supplemental material available online with this article; https://doi.org/10.1172/jci.insight.140169DS1). As such, TP-0903 can better be recognized as a multikinase inhibitor.

Since TP-0903 was shown to inhibit FLT3, we evaluated that activity of TP-0903 against FLT3-ITD and tyrosine kinase domain (TKD) mutants. In a binding assay, TP-0903 showed potent and similar inhibition of FLT3-ITD and TKD mutants, with K_D_ values ranging from 0.79 to 5.6nM (Supplemental Table 1 and Supplemental Figure 1B). For comparison, binding affinities of gilteritinib, midostaurin, and additional tyrosine kinase inhibitors (TKIs) are summarized in Supplemental Table 1. To determine the effects of TP-0903 on the enzymatic activity of FLT3, a kinase assay was performed. TP-0903 inhibited FLT3-ITD and FLT3 D835Y at inhibitory concentrations (IC_50_) of 3.9 and 0.12 nM, respectively (Supplemental Figure 1C).

We further performed in situ quantitative kinase profiling in *FLT3*-ITD–mutated MOLM13 AML cells treated with TP-0903. Using this technique, we observed moderate to strong inhibition of kinases such as AURKA/B/C, CDK6/7, BTK, and JAK1 by TP-0903 100 nM compared with DMSO-treated cells (Figure 1B and Supplemental Table 2). Of note, this assay was unable to detect FLT3 in MOLM13 cells. By immunoblot, TP-0903 inhibited phospho-FLT3 and phospho-STAT5 in MOLM13 and *FLT3*-ITD–mutated MV4-11 cells, as well as ERK/AKT and downstream S6K/S6RP signaling in MOLM13 cells (Figure 1C and Supplemental Figure 2). Consistent with the in situ kinase analysis, TP-0903 also inhibited pAURKA/B in MOLM13 cells.

### TP-0903 is active in FLT3-mutated AML.

**Figure 2:**
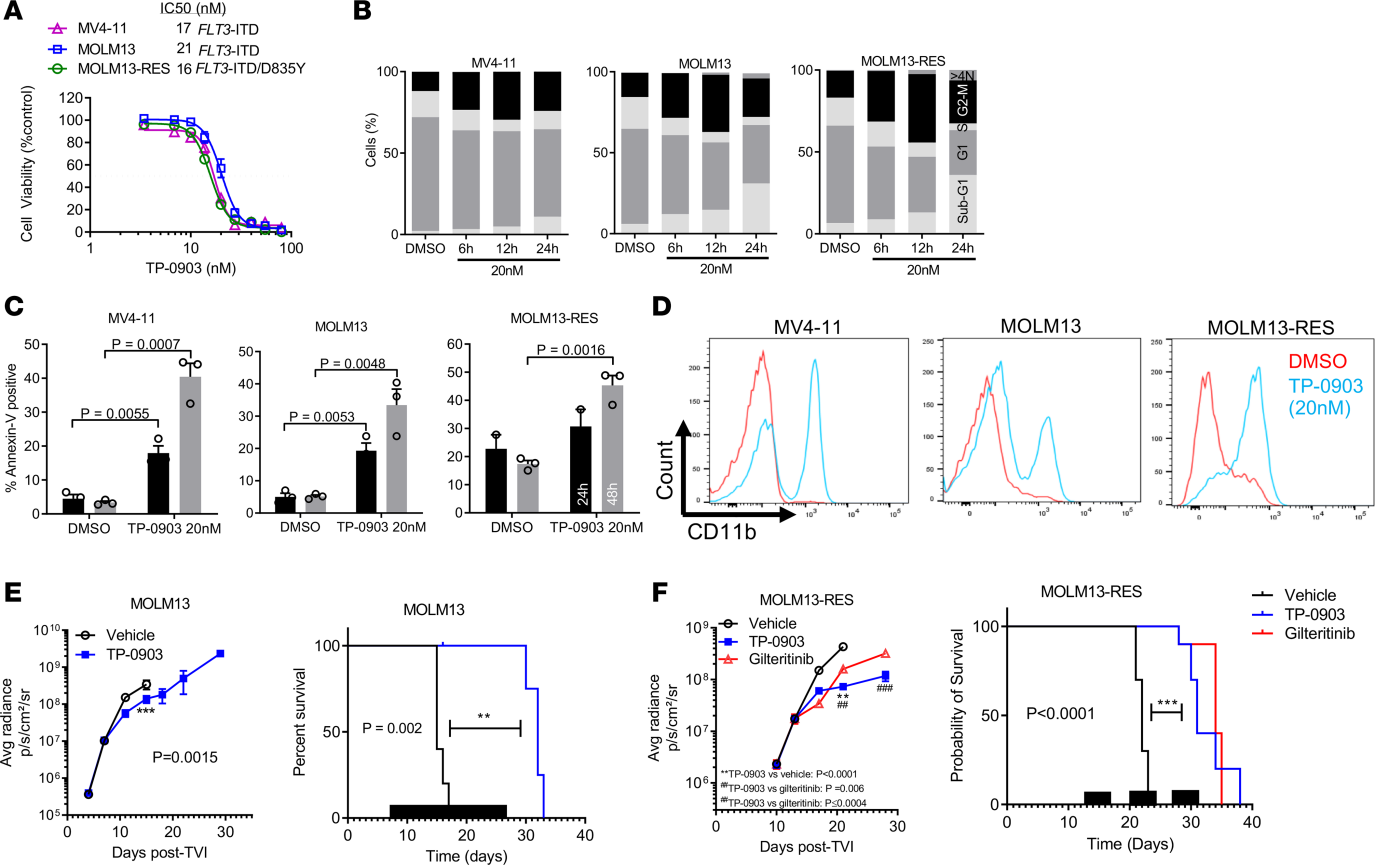
TP-0903 is active in *FLT3*-mutated AML. (**A**) Activity of TP-0903 in FLT3-mutated AML cell lines (MTT assay, 72 hours, *n* = 18). The IC_50_ and mutations present in each cell line are listed. (**B** and **C**) Quantification of cell cycle phase (**B**) and mean (± SEM) annexin-V–positive cells (**C**) at the designated treatment times with TP-0903 (20 nM; *n* = 3). (**D**) Cell differentiation measured by expression of CD11b after treatment with TP-0903 (20 nM) for 72 hours. (**E** and **F**) Bioluminescence signal (mean ± SEM) (left panels) and survival (Kaplan-Meier analysis) (right panels) following treatment with TP-0903 (60 mg/kg), gilteritinib (30 mg/kg), or vehicle in MOLM13-Luc^+^ (*n* = 5/cohort) and MOLM13-RES-Luc^+^ (*n* = 10/cohort) NSG mouse xenograft models. Black bars depict treatment days. *P* values were determined using either unpaired 2-tailed Student’s *t* test (**C** and **E**), 1-way ANOVA (*P* < 0.0001; **F**) with Tukey multiple comparison test or log rank test (**F**; survival curve). Specific *P* values are indicated within the figure.

To fully investigate its therapeutic potential in *FLT3*-ITD^+^ AML, we assessed TP-0903 in *FLT3*-ITD^+^ AML cell lines including MOLM13, MV4-11, and MOLM13-RES. MOLM13-RES cells are a FLT3 TKI–resistant progeny of MOLM13 cells that harbor a dual ITD/D835Y mutation (21). In a viability assay, TP-0903 decreased the growth of MV4-11, MOLM13, and MOLM13-RES cells with similar IC_50_ values (range, 16–21 nM) (Figure 2A). For comparison, IC_50_ values for TP-0903 and additional TKIs are summarized in Supplemental Table 3.

To elucidate how TP-0903 exerts antileukemic activity in *FLT3* mutated AML, we performed cell cycle, apoptosis, and differentiation assays in MV4-11, MOLM13, and MOLM13-RES cells. TP-0903 (20 nM) induced a G2-M cell cycle arrest at 12 hours (suggesting AURKA inhibition), polyploidy (>4N) at 24 hours (suggesting AURKB inhibition), and significant apoptosis at 24 and 48 hours of treatment (*P* < 0.01) in all cell lines (Figure 2, B and C, and Supplemental Figure 3, A and B). Since FLT3 inhibitor therapy has been reported to induce terminal differentiation of leukemia cells (22–24), we determined the effects of TP-0903 on AML cell differentiation. Treatment with TP-0903 (20 nM) for 72 hours increased cell surface expression of CD11b in all cell lines, suggesting induction of differentiation by TP-0903 (Figure 2D). TP-0903 also increased the expression of lysozyme and GCSFR, further supporting cellular differentiation (Supplemental Figure 3C). Using a CFU assay, we also determined the effects of TP-0903 on the proliferation and differentiation of an *FLT3*-ITD mutant primary leukemia sample. Compared with DMSO treatment, TP-0903 (20 nM and 50 nM) inhibited colony formation and induced multinucleation of primary samples, further supporting that TP-0903 is able to differentiate cells (Supplemental Figure 3D).

With positive in vitro data, we then evaluated the in vivo therapeutic potential of TP-0903 in a MOLM13-Luciferase (MOLM13-Luc^+^) xenograft model. We performed tolerability studies in tumor-bearing 8- to 12-week-old female NOD-scid-γ (NSG) mice. It was determined that TP-0903 (40 and 60 mg/kg) were the highest tolerated doses when administered once daily continuously or for 5 consecutive days per week for up to 3–6 weeks (Supplemental Figure 4). Plasma exposure achieved in mice at TP-0903 (40–60 mg/kg) is in the range observed in patients enrolled to the ongoing phase I trial (personal communication, Sumitomo Dainippon Pharma [SDP] Oncology). For efficacy studies, treatments began on day 7, after sufficient engraftment in bone marrow was documented by bioluminescence imaging (Figure 2E). TP-0903 administered at 2 different schedules (60 mg/kg once daily continuously or 5 days a week) for 3 weeks suppressed leukemia outgrowth, as assessed by bioluminescence imagining, and prolonged survival by 17 and 13 days, respectively, compared with vehicle-treated mice (*P* = 0.002 and *P* < 0.0001) (Figure 2E and Supplemental Figure 5 and 6).

The in vivo efficacy of TP-0903 and gilteritinib were then evaluated in a model of drug-resistant *FLT3*-ITD–mutated AML. In a MOLM13-RES-Luc^+^ cell xenograft model in NSG mice, TP-0903 (60mg/kg) or gilteritinib (30 mg/kg) were administered once daily for 5 days/week for 3 weeks. Leukemic cell outgrowth was significantly reduced in mice treated with TP-0903 compared with vehicle or gilteritinib (*P* < 0.0001 and *P* ≤ 0.0004, respectively) (Figure 2F and Supplemental Figure 7). Both TP-0903 and gilteritinib treatment produced a significant increase in survival compared with vehicle-treated mice (median survival: 31, 34, and 22 days for TP-0903–, gilteritinib-, and vehicle-treated groups, respectively; *P* < 0.0001) (Figure 2F).

### TP-0903 is active against the TKI-resistant FLT3 F691L gatekeeper mutation.

**Figure 3:**
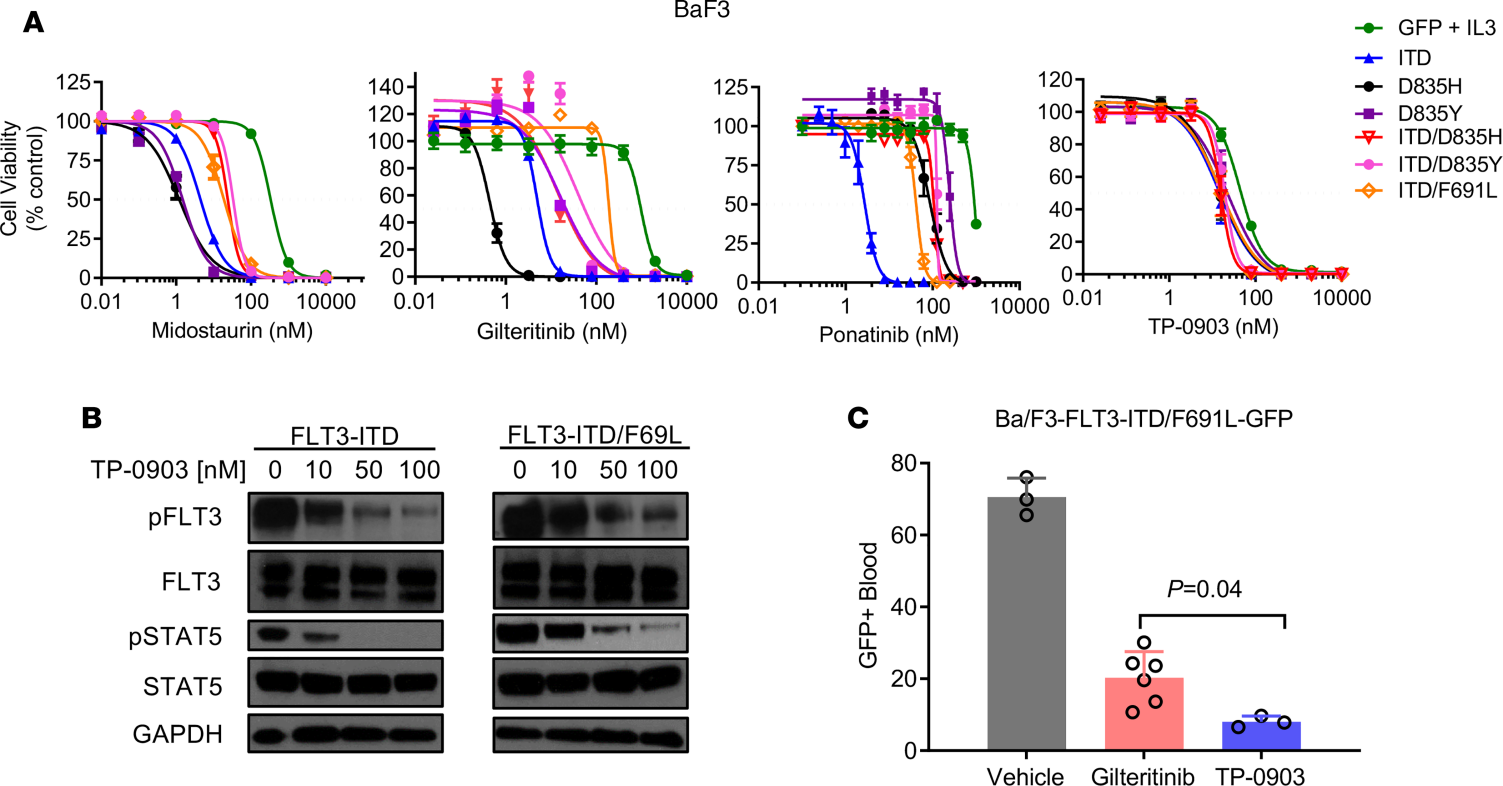
TP-0903 is active against the TKI-resistant *FLT3* F691L gatekeeper mutation. (**A**) Inhibition of viability of Ba/F3 cells expressing FLT3 mutants when treated with indicated TKI (MTT assay, 72 hours, *n* = 18). (**B**) Signaling inhibition in indicated Ba/F3 cells treated with DMSO or increasing concentrations of TP-0903 for 1 hour. Western blot analysis was performed on whole-cell lysates run on parallel gels with the indicated antibodies and is representative of 2–3 independent experiments. (**C**) Peripheral blood GFP^+^ cells following treatment TP-0903 (60 mg/kg) or gilteritinib (30 mg/kg) once daily (starting at day 3) in a Ba/F3-*FLT3*-ITD/F691L-GFP^+^ NSG mouse xenograft model (*n* = 3–6/cohort). GFP^+^ cells were measured via flow on day 15. *P* values were determined using 1-way ANOVA (*P* < 0.0001) with Tukey multiple comparison test, with specific *P* values indicated within the figure.

One mechanism that promotes clinical resistance to FLT3 TKIs is the emergence of the *FLT3* TKD F691L gatekeeper mutation (10, 14). Using murine pro-B Ba/F3 cells expressing *FLT3*-ITD and -ITD/TKD mutants as a drug-resistance model, we evaluated the activity of TP-0903 and a panel of TKIs that inhibit FLT3-ITD. TP-0903 showed similar IC_50_ values in Ba/F3 cells expressing FLT3-ITD (15 nM), D835H/Y (16–22 nM), or F691L (16 nM) mutants. Cells with FLT3-ITD/F691L versus FLT3-ITD showed mild resistance to midostaurin and ponatinib (4.8- and 15-fold, respectively), moderate resistance to crenolanib and gilteritinib (32- and 36-fold, respectively), and strong resistance to sorafenib and quizartinib (956- and 1547-fold, respectively) (Figure 3A and Supplemental Table 3). In parallel, we also observed inhibition of phospho-FLT3 and downstream phospho-STAT5 in Ba/F3-*FLT3*-ITD/F691L cells when they were treated with increasing concentrations of TP-0903. We next evaluated the in vivo activity of TP-0903 and gilteritinib in a Ba/F3 *FLT3*-ITD/F691L-GPF^+^ cell xenograft model (Figure 3B). Compared with gilteritinib treatment, TP-0903–treated mice had a significantly lower peripheral blood GFP signal after 13 days of treatment (*P* = 0.02) (Figure 3C). Overall, these data suggest that TP-0903 may be beneficial in the context of gatekeeper mutant *FLT3*-ITD^+^ AML.

### TP-0903 overcomes resistance due to bone marrow microenvironment–mediated factors.

**Figure 4:**
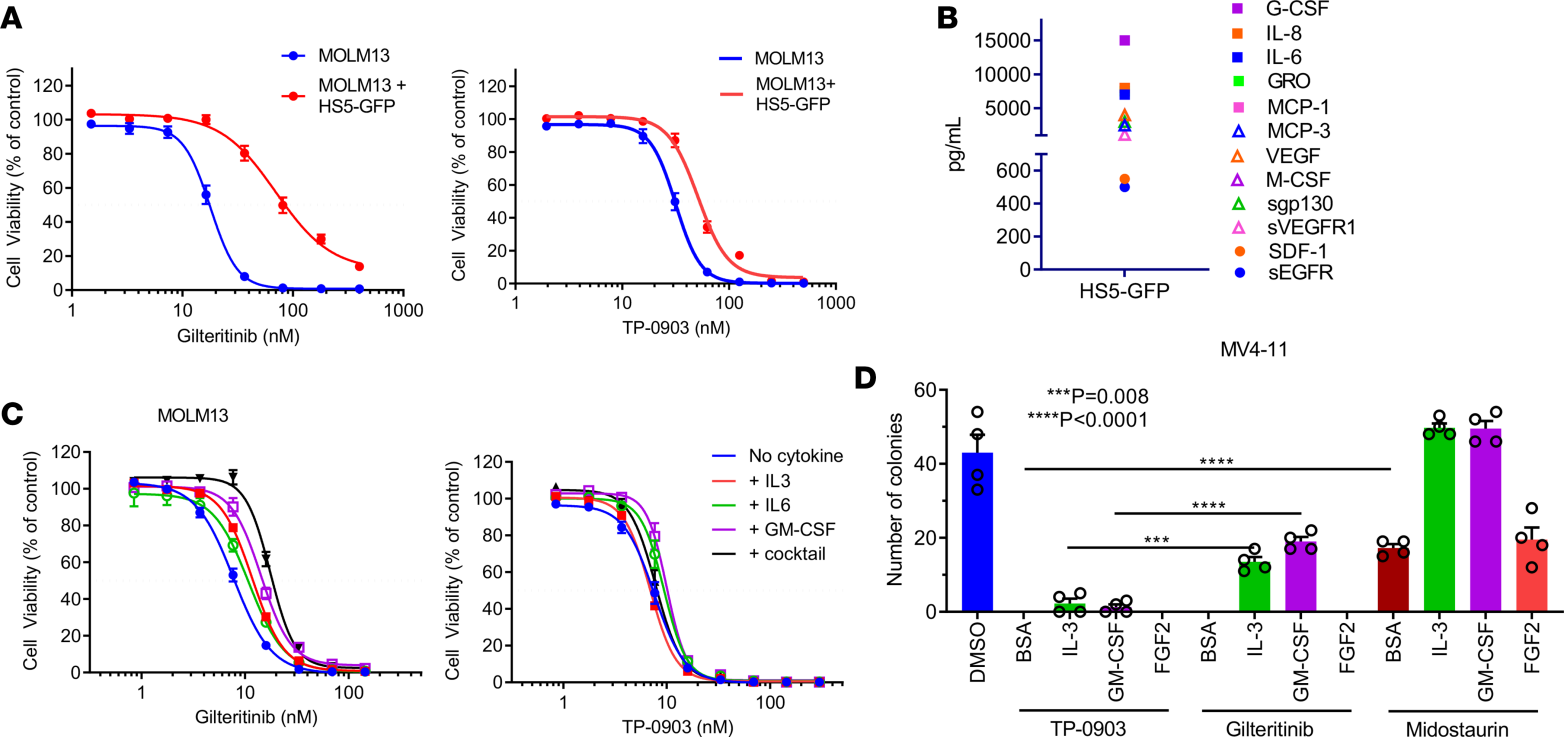
TP-0903 overcomes drug resistance due to bone marrow microenvironment–mediated factors. (**A**) Inhibition of MOLM13 cell viability ± coculture with HS5-GFP MSCs (MTT assay, 72 hours, *n* = 24). (**B**) Cytokine/chemokines/growth factors secreted by HS5-GFP cells (Luminex multiplex assay, *n* = 2). (**C** and **D**) Growth inhibition of MOLM13 (MTT assay, 72 hours, *n* = 12) (**C**) or 7-day MV4-11 CFU assay (*n* = 4) (**D**) treated with indicated TKI ± cytokine/chemokine/growth factor support. *P* values were determined using 1-way ANOVA (*P* < 0.0001) with Tukey multiple comparison test, with specific *P* values indicated within the figure.

Recent preclinical studies indicate that *FLT3*-ITD–mutated AML cells are rescued from FLT3 inhibition due to bone marrow microenvironment–mediated factors including stromal cells, involving direct cell-cell contact and soluble factors, as well as hematopoietic cytokines and growth factors (e.g., FLT3 ligand, AXL, IL-3, GM-CSF, and FGF2) (25–29). Coculture of MOLM13 cells with HS5-GFP human bone marrow stromal cells (MSCs) induced a minimal 1.7-fold right shift in the TP-0903 IC_50_ value compared with MOLM13 cells in culture alone, whereas a 4.9- and 2.5-fold right shift in IC_50_ value was observed for gilteritinib and midostaurin, respectively (Figure 4A and Supplemental Figure 8A). For comparison, inhibition of the viability of MOLM13 and HS5-GFP cells alone in coculture following treatment with TP-0903 and additional TKIs are shown in Supplemental Figure 8A. In culture, HS5-GFP secretes a number of cytokines, chemokines, and growth factors that likely contribute to TKI resistance in MOLM13/MSC coculture (Figure 4B). To test this, MOLM13 cells were treated with TP-0903, gilteritinib, or midostaurin in the presence of IL-3, IL-6, GM-CSF, or a cocktail consisting of all 3. We observed minimal changes (1.3-fold) in TP-0903 IC_50_ values in the presence of cytokines/growth factors, whereas with gilteritinib and midostaurin, we observed an IC_50_ value shift upward of 3-fold (Figure 4C and Supplemental Figure 8B). In a CFU assay with MV4-11 cells supplemented with IL-3, GM-CSF, or FGF2, which was shown previously to induce resistance to the FLT3 TKI quizartinib (27), TP-0903 significantly inhibited colony formation under all conditions. In contrast, IL-3 and GM-CSF partially rescued cells during gilteritinib treatment, while IL-3, GM-CSF, and FGF2 fully or partially rescued cells during midostaurin treatment (Figure 4D). These data demonstrate that TP-0903 is able overcome drug resistance due to several bone marrow microenvironment–mediated factors, which is a short-coming of US Food and Drug Administration–approved (FDA-approved) and clinical candidate FLT3 TKIs.

### TP-0903 is active in models of AML with recurrent mutations.

**Figure 5:**
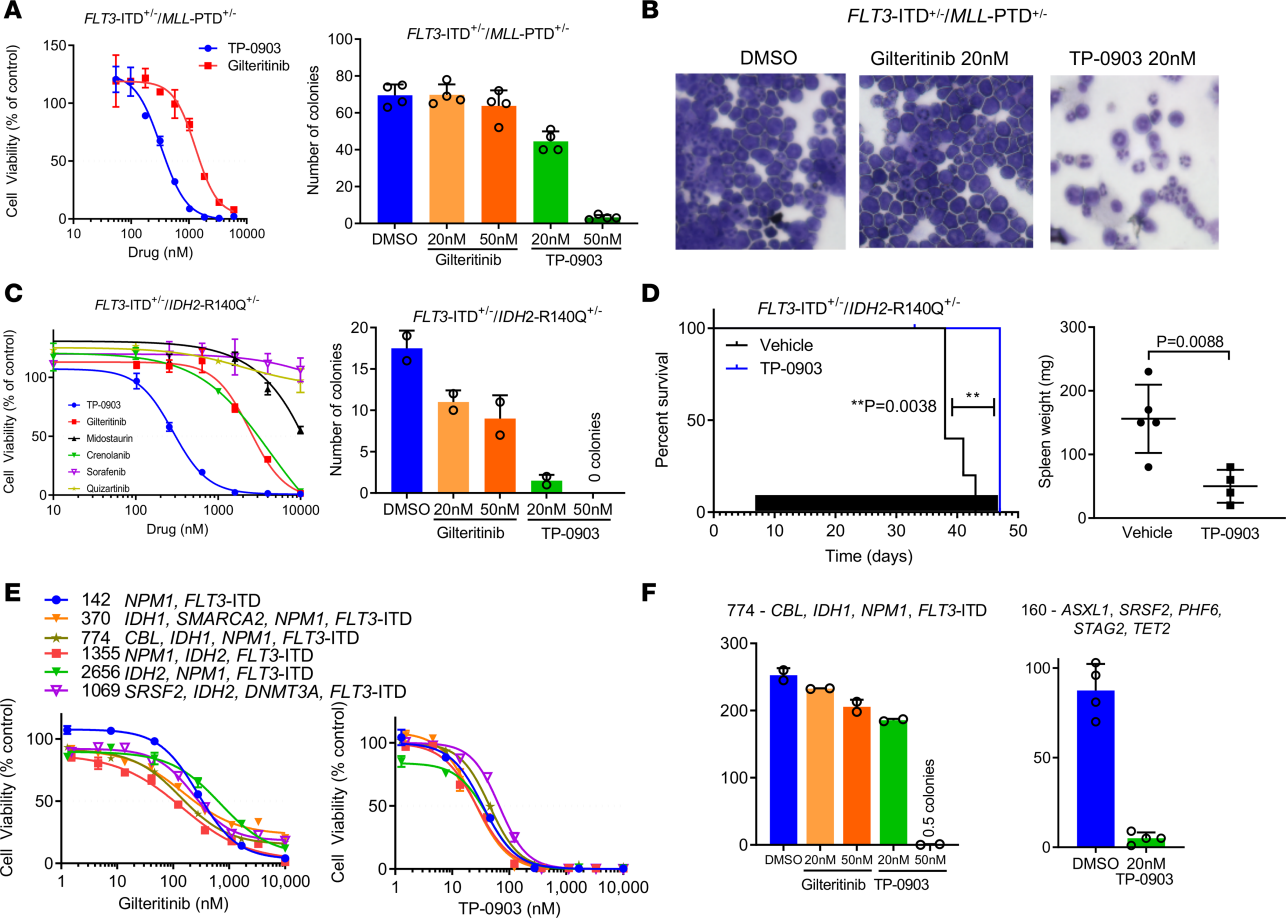
TP-0903 is active in models of AML with recurrent mutations. (**A**) Inhibition of cell viability (CellTiterGlo assay, 72 hours, *n* = 4) (left panel) and colony formation (7 day CFU assay, *n* = 4) of primary *FLT3*-ITD/*MLL*-PTD murine leukemia cells treated ex vivo with indicated TKI. (**B**) Morphology of treated cells at day 7 by Wright Giemsa staining. Total magnification, 400×. (**C**) Inhibition of cell viability (CellTiterGlo assay, 72 hours, *n* = 3–6) and colony formation (7 day CFU assay, *n* = 4) of primary *FLT3*-ITD/*IDH2*-R140Q murine leukemia cells treated ex vivo with indicated TKI. (**D**) Survival by Kaplan-Meier analysis (left panel) and spleen weight at end of study (right panel) following treatment with TP-0903 (40 mg/kg) once daily or vehicle (*n* = 5/cohort). Black bars depict days of treatment. (**E**) Inhibition of viability of human primary AML samples with *FLT3*-ITD and indicated co-occurring mutations treated with indicated TKI ex vivo (CellTiterGlo assay, 72 hours, *n* = 3). (**F**) Inhibition of colony formation of *FLT3*-ITD^+^ human primary AML samples treated with indicated TKI (14 day CFU assay, *n* = 2). *P* values were determined using either unpaired 2-tailed Student’s *t* test or log rank test. Specific *P* values are indicated within the figure.

In AML, previous studies have reported recurrent mutations in genes such as *ASXL1*, *DNMT3A*, *FLT3*-ITD, *IDH1/2*, NPM1, *TET2*, *WT1*, and *MLL*-PTD, which impact prognosis and clinical outcomes (1, 12, 13). More recently, several investigations have established the contribution of clonal heterogeneity and co-occurring mutations to response to targeted agents (10, 14). Thus, we set out to investigate the activity of TP-0903 in models of co-occurring recurrent mutations. Using AML samples from a genetically modified *FLT3*-ITD^+/–^ *MLL*-PTD^+/–^ double knock-in mouse model (11), ex vivo treatment with TP-0903 was performed. In a viability assay, cells were 3.8-fold more sensitive to TP-0903 than gilteritinib (Figure 5A). Similarly, in a CFU assay, TP-0903 (20 nM and 50 nM) inhibited colony formation, whereas gilteritinib showed no activity at the same concentrations (Figure 5A). Morphological assessment demonstrated that TP-0903 induced cellular differentiation of *FLT3*-ITD^+/–^ *MLL*-PTD^+/–^ cells, as indicated by maturation with multilobulated nuclei, which was not observed with gilteritinib (Figure 5B). We then compared ex vivo inhibition activity of TP-0903 on AML samples from a *FLT3*-ITD^+/–^ *IDH2*-R140Q^+/–^ double knock-in mouse model (30, 31). In a viability assay, TP-0903 was greater than 8-fold more potent than gilteritinib and midostaurin; in a CFU assay, TP-0903 (20 nM and 50 nM) inhibited colony formation, whereas gilteritinib had modest activity (Figure 5C). IC_50_ values for inhibition of *FLT3*-ITD^+/–^ *IDH2*-R140Q^+/–^ cell viability by TP-0903 and additional TKIs are summarized in Supplemental Table 3.

With positive ex vivo results, we then evaluated the in vivo activity of TP-0903 in a primary transplantation model of murine *FLT3*-ITD^+/–^ *IDH2*-R140Q^+/–^ AML cells in NSG mice. Starting on day 7 after transplantation, mice were treated with TP-0903 (40 mg/kg) once daily for up to 6 weeks. TP-0903 prolonged survival by 9 days compared with vehicle-treated mice (*P* = 0.0038). At study endpoint, TP-0903 treatment also resulted in a reduction in spleen weight compared with DMSO-treated mice (mean ± SD, 50 ± 13 mg versus 156 ± 24 mg, respectively; *P* = 0.0088) (Figure 5D).

Given the promising results in murine leukemia models with co-occurring mutations, we then evaluated the ex vivo activity of TP-0903 and TKIs in human primary samples with coexisting recurrent mutations (e.g., *FLT3*-ITD, *IDH*1/2, *ASXL1*, *DNMT3A*, *NPM1*, and *SRSF2*). In all samples, TP-0903 inhibited cell viability at IC_50_ values < 100 nM (median, 35 nM; range, 21–67 nM); in AML samples with *FLT3*-ITD mutations, gilteritinib was less effective with all but 1 IC_50_ value > 100 nM (median, 263 nM; range, 93–1307 nM) (Figure 5E). In reference to other TKIs, these samples either responded similarly to gilteritinib or were less effective, and none exhibited the same potency as TP-0903 (Supplemental Table 3 and Supplemental Figure 9). In a CFU assay, TP-0903 (20–50 nM) effectively suppressed colony formation of primary AML samples, including 1 with a complex set of mutations in epigenetic modifiers and RNA-splicing genes (Figure 5F). These data further support that TP-0903 retains activity in multiple models of AML with co-occurring mutations that confer drug resistance.

### TP-0903 is active in AML with RAS pathway mutations.

**Figure 6:**
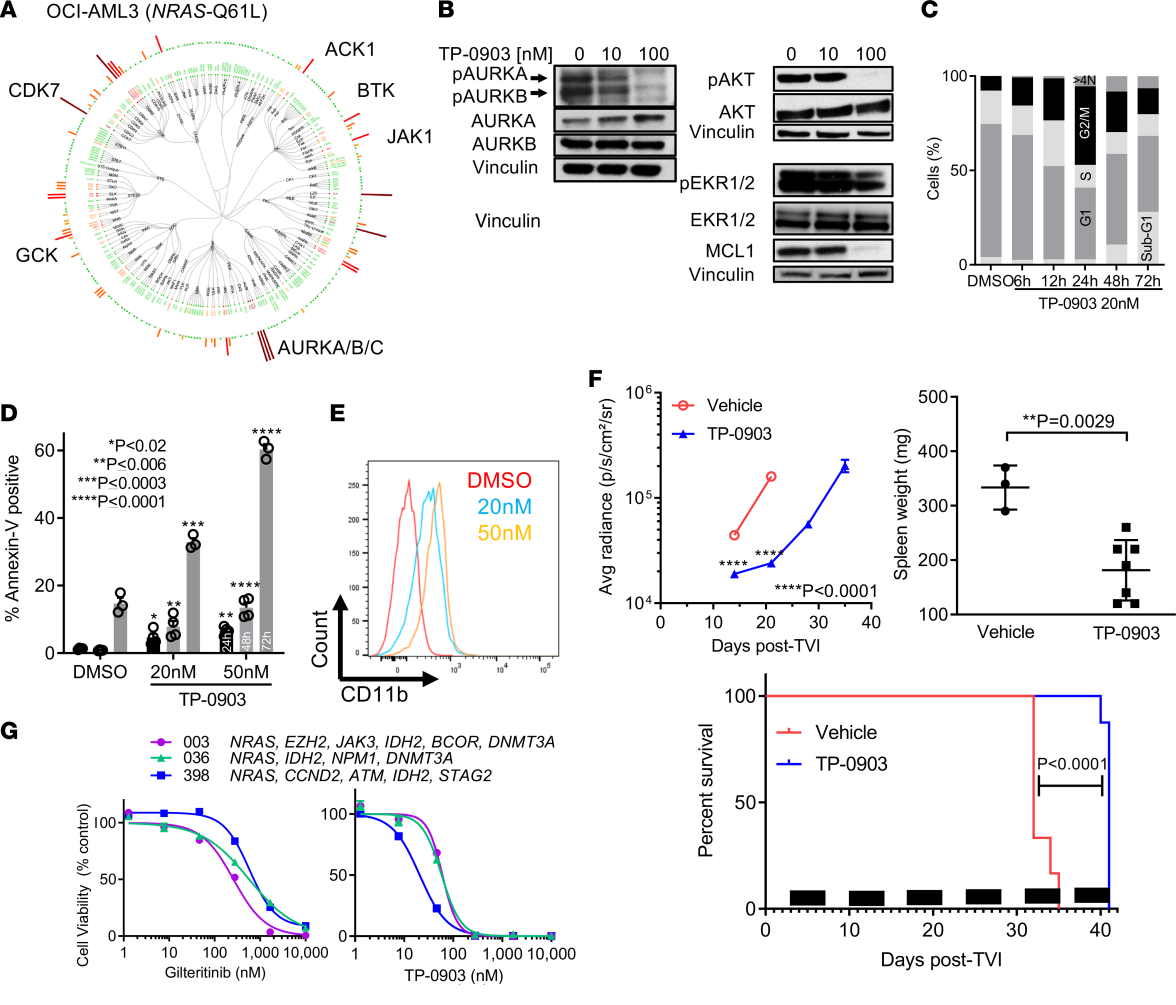
TP-0903 is active in AML with *RAS* pathway mutations. (**A**) Dendogram of native kinase inhibition in OCI-AML3 cells treated with 100 nM TP-0903 for 2 hours in a KiNativ assay. All kinases detected are shown and grouped based on classification. Inhibition is relative to the DMSO control indicated on a spectrum of dark red (>90%), red (75%–90%), orange (50%–75%), yellow (35%–50%), and green (no change) bars. (**B**) Signaling inhibition of OCI-AML3 cells treated with DMSO or increasing concentrations of TP-0903 for 4 hours. Western blot analysis was performed on whole-cell lysates run on parallel gels with the indicated antibodies and is representative of 2 independent experiments. (**C** and **D**) Quantification of cell cycle phase (**C**) and mean (± SEM) annexin-V–positive cells (**D**) at the designated treatment times with TP-0903 (20 nM) or TP-0903 (50 nM) (*n* = 3). (**E**) Cell differentiation measured by expression of CD11b after treatment with TP-0903 (20 nM and 50 nM) for 72 hours (representative images). (**F**) Bioluminescence signal (mean ±SEM) and survival (Kaplan-Meier analysis) and spleen weight at study end following treatment with TP-0903 (50 mg/kg) once daily (*n* = 6) or vehicle (*n* = 8) in an OCI-AML3–Luc^+^ NSG mouse xenograft model. Black bars depict treatment days. (**G**) Inhibition of viability of human primary AML samples with indicated mutations treated with indicated TKI (CellTiterGlo, 72 hours, *n* = 3). *P* values were determined using either unpaired 2-tailed Student’s *t* test (**F**), 1 way ANOVA (*P* < 0.002; **D**) with Dunnett’s multiple comparison test, or log rank test (**F**; survival curve). Specific *P* values are indicated within the figure.

Recent publications demonstrated that *RAS* pathway mutations contribute to clinical resistance to the FLT3 TKIs gilteritinib and crenolanib (10, 14). Therefore, we evaluated the activity of TP-0903 in models of *NRAS* mutant AML. In OCI-AML3 cells with a *NRAS*-Q61L mutation, TP-0903 inhibited viability with an IC_50_ value of 37 nM. To obtain insight into potential mechanisms of action of TP-0903, we determined the native kinases inhibited in OCI-AML3 cells treated with TP-0903 (100 nM). We observed similar inhibition of kinases in OCI-AML3 as with MOLM13 such as AURKA/B/C, BTK, CDK7, and JAK1 (Figure 6A and Supplemental Table 4). Interestingly, we observed 87% inhibition of ACK1 (activated CDC42 kinase 1, also known as TNK2) and 46% inhibition of GCK (germinal center kinase, also known as MAP4K2) kinases. In a previous investigation, an integrated approach involving cell-based pharmacologic screening combined with native kinase profiling, gene expression profiling, and mechanism studies, identified ACK1 and GCK kinases that synergistically contributed to the growth of NRAS transformed leukemia cells, including OCI-AML3 cells (32). This suggested that inhibition of these kinases may be involved in TP-0903 activity in *RAS* mutant AML. In biochemical assays, we confirmed that TP-0903 potently inhibits ACK1 (K_D_ = 7.3 nM, IC_50_ = 30.7 nM) and GCK (K_D_ = 1.8 nM, IC_50_ = 2.5 nM) (Supplemental Figure 10). By immunoblot, we showed that TP-0903 (100 nM) inhibited pAURKA, pAURKB, pAKT, and MCL1 (Figure 6B). In subsequent studies in OCI-AML3 cells, TP-0903 (20 nM) induced a G2-M cell cycle arrest at 12–24 hours and polyploidy at 24 hours, and significant apoptosis was observed after 72 hours of treatment with 20–50 nM (P ≤ 0.0009) (Figure 6, C and D). Furthermore, we observed an increase in CD11b expression, indicating cellular differentiation by TP-0903 (Figure 6E).

With positive in vitro data, we evaluated the in vivo activity of TP-0903 in a OCI-AML3–Luc^+^ mouse xenograft model. Starting 3 days after cell injection, mice were treated with TP-0903 (50 mg/kg) once daily for 5 days/week for up to 6 weeks. Compared with vehicle-treated mice, TP-0903 suppressed the outgrowth of leukemia cells (*P* < 0.0001) and prolonged survival by 9 days (*P* < 0.0001) (Figure 6F and Supplemental Figure 11). Furthermore, at study endpoint, TP-0903 treatment resulted in a reduction in spleen weight compared with vehicle control (mean ± SD, 181 ± 21 mg versus 333 ± 23 mg versus, respectively (*P* = 0.0029) (Figure 6F).

Given the encouraging results in OCI-AML3 cells, we compared the ex vivo activity of TP-0903 and gilteritinib in human primary samples with *RAS* (*NRAS*, *KRAS*, and *PTPN11*) mutations, along with other mutations frequently found in patients representing the clonal heterogeneity observed in AML. In these samples, TP-0903 inhibited viability with IC_50_ values ranging from 21 to 60 nM, whereas these mutations rendered the cells highly resistant to gilteritinib with IC_50_ values ranging from 260 to 731 nM (Figure 6G).

**Figure 7:**
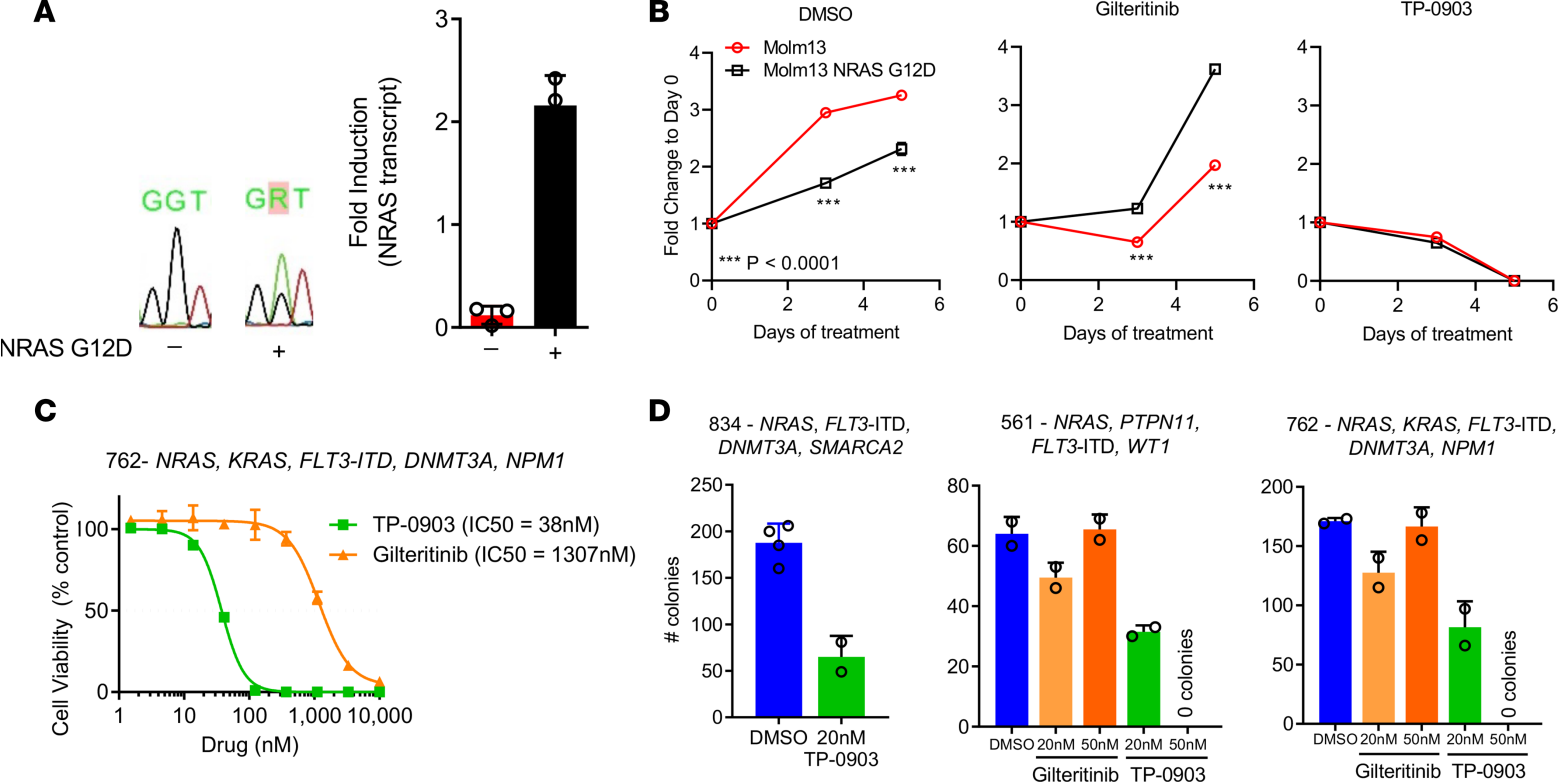
TP-0903 is active in AML with co-occurring *FLT3*-ITD and *NRAS* mutations. (**A**) Sanger sequencing confirmation of the NRAS G12D mutation and RT-PCR showing increase NRAS expression after 24 hours of induction with 0.1 μg/mL doxycycline in MOLM13 cells with (+) and without (–) *NRAS* G12D. (**B**) Cell growth of MOLM13 and MOLM13 *NRAS* G12D when treated with DMSO or 15 nM of TP-0903 or gilteritinib induction with 0.1 μg/mL doxycycline (*n* = 6, representative of 3 independent experiments). (**C** and **D**) Inhibition of viability (CellTiterGlo, 72-hour treatment, *n* = 3) (**C**) or colony formation (14-day CFU assay, *n* = 2) (**D**) of human primary AML samples with *FLT3*-ITD and *RAS* pathway mutations treated with TKI indicated. *P* values were determined using unpaired 2-tailed Student’s *t* test and are indicated within the figure.

We next evaluated the activity of TP-0903 against *FLT3*-ITD in the context of *NRAS* mutations. We generated a stably transduced *FLT3*-ITD MOLM13 cell line carrying an inducible *NRAS* G12D mutation (MOLM13 *NRAS* G12D). Cells underwent Sanger sequencing to confirm the presence of the *NRAS* G12D mutation, and expression of NRAS transcript was confirmed by real-time RT-PCR (Figure 7A). When MOLM13 *NRAS* G12D cells were grown in the absence of drug treatment (DMSO), we saw a growth disadvantage compared with the MOLM13 parental cell line. This growth discrepancy is similar to what was observed previously in a MOLM14 cell line carrying an *NRAS* mutation (14). When MOLM13 parental or MOLM13 *NRAS* G12D cells were treated with TP-0903 (15 nM), we observed inhibition of cell growth at day 3 and complete loss of viable cells by day 5. In contrast, when MOLM13 *NRAS* G12D cells were treated with gilteritinib (15 nM), a resistant phenotype was observed with outgrowth of cells, as previously reported (Figure 7B) (14). We then compared the ex vivo activity of TP-0903 and gilteritinib in 3 human primary AML samples with *FLT3*-ITD and co-occurring *RAS* pathway (*NRAS*, *KRAS*, and *PTPN11*) mutations. TP-0903 was 34-fold more potent than gilteritinib in inhibiting cell viability with IC_50_ values of 38 nM and 1307 nM, respectively (Figure 7C). Similarly, in a CFU assay, TP-0903 (20–50 nM) reduced colony formation of 3 primary samples, whereas gilteritinib had no activity (Figure 7D). Although single-cell DNA sequencing was not performed in these primary samples to confirm the coexistence of *FLT3*-ITD and *RAS* pathway mutations in the same AML cells, this data further support that TP-0903 is able to overcome drug resistance observed by gilteritinib when *NRAS* mutations are present.

### Effect of TP-0903 on human normal cells.

To determine the toxicity of TP-0903 on normal human cells, we evaluated the effects of TP-0903 on cord blood CD34^+^ cells and dermal fibroblasts, each from 2 different donors (Supplemental Figure 12). TP-0903 inhibited colony formation of CD34^+^ cells at a concentration of 200 nM and viability of fibroblasts at concentrations ≥ 250 nM. In addition, TP-0903 was not cytotoxic to HS5 human MSCs at concentrations < 250 nM (Supplemental Figure 8A). TP-0903 concentrations producing toxicity in normal human cells are 5- to 10-fold higher than concentrations shown to exert antileukemic activity in AML cell lines and primary AML samples (20–50 nM). These data demonstrate that the preclinical antileukemic activity of TP-0903 is achieved below exposures associated with toxicity to normal cells.

## Discussion

Clonal heterogeneity remains one of the biggest challenges in AML therapy due to variable responses contributed by recurrent molecular alterations. Responses to targeted agents are generally not sustained due to resistance in the presence of multiple co-occurring mutations and the evolution of polyclonal drug resistance associated with diverse molecular alterations (10, 14, 33–35). In this present study, we demonstrate that TP-0903 has broad therapeutic potential in not only *FLT3*-ITD^+^ AML, but also in AML, with recurrent comutations that are associated with poor prognosis or confer drug resistance. TP-0903 retained activity in AML with *FLT3*-ITD and TKD mutations — including the F691L gatekeeper mutation, which promotes varying degrees of resistance to current FLT3 TKIs — and was able to overcome FLT3 TKI resistance due to bone marrow microenvironment–mediated stromal cell, cytokine, chemokine, and growth factor support. In addition, TP-0903 retained activity in multiple models of AML with co-occurring recurrent mutations.

From kinome profiling, TP-0903 displayed multikinase inhibitor properties, which may be an attractive approach in a molecularly heterogeneous disease such as AML. TP-0903 has been well tolerated in the ongoing clinical trials in solid tumors and CLL (CinicalTrials.gov; NCT02729298 and NCT03572634). We observed that TP-0903 induced G2-M cell cycle arrest, polyploidy, and apoptosis. The G2-M arrest is most likely due to the inhibition of AURKA kinase, while polyploidy can be attributed to the inhibition of AURKB kinase; additional effects on cell cycle regulation may be mediated through inhibition of CDKs, CHEK1/2, and PLK4 (36–41). We hypothesize that multikinase inhibition may contribute to its potent antileukemic activity across different recurrent somatic mutations. Leukemic cells treated with TP-0903 underwent differentiation, as determined by increased CD11b expression and morphological maturation with multilobulated nuclei, suggesting an additional mechanism of action may be through the induction of cellular differentiation.

We demonstrated that TP-0903 has potent activity against de novo and drug-resistant *FLT3*-ITD^+^ AML. While the *FLT3* F691L gatekeeper mutation promotes varying degrees of resistance to current FLT3 TKIs in preclinical models and has been shown to emerge at resistance during treatment with several of these agents (10, 14, 23, 35, 42, 43), TP-0903 maintained near-equipotent activity in cells expressing this mutation. How TP-0903 retains potency against the gatekeeper mutation is unknown. However, TP-0903 was originally designed to bind to the AXL gatekeeper residue L671 (17). The leucine substitution in FLT3 may exhibit the same properties as L671 in AXL and may explain the activity of TP-0903 in *FLT3*-ITD/F691L–mutated AML. In in vitro models recapitulating the bone marrow microenvironment, TP-0903 retained activity where other FLT3 TKIs, including gilteritinib and midostaurin, fell short and were less active. The ability of TP-0903 to remain active in the context of *FLT3* F691L mutation, as well as resistance rendered by cytokine support mediated by the bone marrow microenvironment, may provide benefit over gilteritinib and other FLT3 TKIs. We postulate that TP-0903 overcomes these resistance mechanisms, in part, based on its unique multikinase inhibitor profile and multiple modes of action.

In patients with *FLT3*-mutated relapsed/refractory disease treated with crenolanib, a high frequency of mutations in RAS pathway (*NRAS*, *PTPN11*, *KRAS*, and *CBL*), *IDH1*, *TET2*, and *DNMT3A* were present in poor responders and were not cleared during treatment with new *NRAS* mutations emerging in several patients (10). In a study of baseline and progression samples from gilteritinib-treated patients, single-cell–targeted DNA sequencing revealed the emergence of RAS (*NRAS*, *KRAS*, *PTPN11*) and *IDH2* mutations. Baseline *NPM1*, *DNMT3A*, *IDH1/2*, *WT1*, *ASXL1*, and more mutations were still detected at the end of gilteritinib therapy (14). In *IDH2* mutant patients treated with enasidenib, nonresponders had significantly more co-occurring mutations, and notably, *RAS* pathway mutations attenuated response to enasidenib, as a dominant or minor subclone (33). Here, we showed that TP-0903 was active in models of *RAS* pathway mutant AML with or without *FLT3*-ITD and other co-occurring mutations, while these models were highly resistant to gilteritinib treatment. Through kinome profiling, we found that TP-0903 inhibited both ACK1 and GCK kinases, which have been reported to synergistically contribute to the growth of *NRAS* mutant leukemia cells (32). It is known that *NRAS* mutations lead to constitutive RAS activation and subsequent activation of multiple signaling pathways, including the PI3K-AKT (44). Activation of AKT by ACK1, in a PI3K-independent manner, has been reported, and that suppression of this signaling is important to overcome the oncogenic nature of *NRAS* mutations (32, 45). After treatment with TP-0903, we measured a decrease in AKT signaling, which is likely a result, in part, of inhibition of ACK1. Taken together, it is likely that inhibition of ACK1 and GCK kinases contributes to the activity of TP-0903 in *NRAS* mutant leukemia cells. Furthermore, the observation that TP-0903 retains activity in *NRAS* mutant AML, irrespective of *FLT3*-ITD status, merits further investigation into the activity of TP-0903 in other forms of drug-resistant AML due to *RAS* pathway mutations.

In addition to *RAS* pathway mutations, other genetic abnormalities, such as in *ASXL1*, *SRSF2*, *IDH1/2*, *DNMT3A*, *NPM1*, *MLL*-PTD, *CBL*, *FLT3*, and *BCOR*, have been shown to impact prognosis and clinical outcomes (1, 12, 13). Recently, clonal heterogeneity and co-occurring mutations have been associated with poor prognosis and AML-directed therapy resistance, such as with gilteritinib, crenolanib, and enasidenib, as mentioned above (10, 14, 33). Despite having low occurrence, *MLL*-PTD is associated with unfavorable prognosis, and it co-occurs frequently with *FLT3* mutations, which presents as an even poorer prognosis (11). In a genetically engineered double knock-in primary murine AML model, TP-0903 showed potent activity compared with gilteritinib in the presence of *FLT3*-ITD/*MLL*-PTD dual mutations. In the *FLT3*-ITD^+/–^ *IDH2*-R140Q^+/–^ primary murine model, TP-0903 showed both promising ex vivo and in vivo activity. Having both mutations often led to no response or relapse to either FLT3 TKIs or IDH inhibitors presenting as drug resistance mechanisms to both classes of drugs (10, 33, 46). However, our murine data confirm the notion that TP-0903 could be utilized as a therapeutic strategy in such drug-resistant AML settings.

Based on our positive results in the primary murine models, we further sought to investigate the activity of TP-0903 in other AML models with multiple recurrent mutations, which pose as one of the most challenging aspects in treating AML. These primary human samples had 2 or more (up to 6) recurrent mutations that confer poor prognosis. Notably, some of these samples had *NPM1*, *DNMT3A*, and *FLT3*-ITD mutations co-occurring, which has a deleterious effect (1); *ASXL1*, *WT1*, and *FLT3* mutations that carry a poor prognosis (1, 4, 15, 16); *BCOR*, *CBL*, *IDH1/2*, and *RAS* mutations that confer drug resistance (10, 14, 33); *SRSF2*, *EZH2*, and *BCOR* mutations that are associated with secondary AML (4); and more. TP-0903 retained near-equipotent activity against all samples, unlike other agents, where resistance was observed. This data further highlight the activity of TP-0903 in AML models with different mutations that contribute to heterogeneity, to which current treatments are vulnerable.

In our collective studies, TP-0903 demonstrated antileukemic activity in cell lines and primary samples at concentrations of 20–50 nM. These concentrations are in the range of average concentrations achieved in vivo in mice following administration of TP-0903 (60 mg/kg), as used in our preclinical studies, as well as concentrations of active TP-0903 moieties achieved at steady-state in humans receiving TP-0903 once daily at the recommended phase 2 dose of 50 mg (personal communication, SDP Oncology). TP-0903 concentrations producing toxicity in normal human CD34^+^ cells, and fibroblasts cells were 5- to 10-fold higher than concentrations shown to exert antileukemic activity. This demonstrates that the preclinical antileukemic activity of TP-0903 is achieved at clinically relevant exposure and below exposures associated with toxicity to normal cells.

In summary, TP-0903 is a multikinase inhibitor with potent activity against multiple drug-resistant models of AML. Most importantly, TP-0903 was potent in inhibiting the growth of all AML models with heterogeneous groups of recurrent mutations. Due to the heterogeneous nature of de novo and relapsed/refractory AML and clonal evolution that occurs during AML therapy, one could speculate that a multikinase inhibitor such as TP-0903 could produce more durable responses compared with current selective targeted therapies (Supplemental Figure 13). To this effect, TP-0903 should have immediate clinical impact in AML, with broader therapeutic potential in drug-resistant AML with multiple recurrent mutations.

## Methods

### Cell culture and reagents.

Human MV4-11 (ATCC), OCI-AML3 (ATCC), MOLM13 (DSMZ), MOLM13-RES (47), mouse Ba/F3 cells (DSMZ), and HS5-GFP (acquired from William Dalton’s lab, Moffitt Cancer Center, Tampa, Florida, USA) were obtained and maintained in RPMI (Thermo Fisher Scientific) with 10% FBS as previously described (21, 47). Primary human fibroblasts from 2 different sources (ATCC and MilliporeSigma) were cultured in Fibroblast Growth Kit-Low Serum Complete Medium (ATCC PCS-201-041).

Antibodies against FLT3 (3462; clone 8F2), phospho-FLT3 (3464; clone 30D4), STAT5 (94025; clone D206Y), phospho-STAT5 (4322; clone D47E7), AKT (4691; clone C67E7), phospho-AKT (4060; clone D9E), ERK1/2 (4695; clone 137F5), phospho-EKR1/2 (4370; clone D.13.14.4E), S6K (2708; clone 49D7), phospho-S6K (97596; clone D5U10), S6RP (2217; clone 5G10), phospho-S6RP (4858; D57.2.2E), pAURKA/B/C (2914; clone D13A11), AURKA (91590; clone D3V7T), AURKB (3094; clone N/A), vinculin (13901; clone E1E9V), GAPDH (5174S; clone D16H11), MCL (39224; clone D5V5L), and HRP-conjugate secondary anti-rabbit (7074 /clone N/A) were obtained from Cell Signaling Technology (CST). Drugs were obtained from the following sources: TP-0903 (Tolero Pharmaceuticals), gilteritinib (ChemieTek), crenolanib (AROG Pharmaceuticals LLC), midostaurin (LC Laboratories), quizartinib (ChemieTek), sorafenib (LC Laboratories), and RG-7388 (MedChemExpress). Human and mouse cytokines were purchased from Peprotech.

### Western blot.

MOM13 cells were serum starved (RPMI plus 0.5% FBS) for 18–24 hours prior to start of drug treatment. For other cells, they were not serum starved. Drug treatments were carried out for 1 or 4 hours, at which time cells were lysed with Cell Lysis buffer (CST) or RIPA buffer (CST) supplemented with protease and phosphatase inhibitors. Cell lysate concentrations were determined by BCA; equal protein concentrations were separated on Bis-Tris 4-12% SDS-polyacrylamide gel with MOPS buffers according to the manufacturer’s instructions (Invitrogen) and transferred to PVDF membranes followed by Western blot analysis using antibodies indicated above. Western blots were developed with either Signal Fire ECL reagent (CST) or SuperSignal West Femto Maximum Sensitivity Substrate (Invitrogen) using Film or Odyssey FC Imaging System (LI-COR Biosciences). Western blots were repeated in 2–3 biological replicate experiments.

### Kinome profiling.

Binding affinities of TP-0903 to kinases were measured by use of the commercially available KdELECT assay (Eurofins DiscoverX), as previously described (47). Kinase assay of TP-0903 was performed by Reaction Biology.

For kinome analysis, MOLM13 and OCI-AML3 cells were treated with DMSO or TP-0903 (100 nM) for 2 hours, washed in PBS, and pelleted. Samples were analyzed by KiNativ in situ kinase profiling by ActivX Biosciences.

### Cell viability assessment.

Drug treatment effects on cell lines were assessed by MTT assay (Roche Diagnostics), as previously described (21). For human and murine primary leukemic blasts cells, CellTiter Glo assay (Promega) was used based on manufacturer’s instructions. Human primary leukemia samples were cultured in RPMI plus 10% FBS with a human cytokine cocktail consisting 10 ng/mL each of SCF, IL-3, GM-CSF, and FLT3 ligand (Peprotech). Murine primary leukemia samples were cultured in RPMI plus 10% FBS with 1× GlutaMax (Thermo Fisher Scientific) and murine cytokine cocktail of IL-3 (10 ng/mL), SCF (10 ng/mL), and GM-CSF (20 ng/mL). Drug treatments were carried out for 72 hours. Details are provided in the Supplemental Methods.

### CFU assay.

For human primary CFU assays, cells were cultured in MethoCult H4035 Optimum without EPO (Stemcell Technologies). For mouse primary CFU assays, cells were directly cultured in MethoCult M3231 (Stemcell Technologies) supplemented with 45 ng/mL of murine IL-6 (mIL-6), mIL-3, and mSCF. Both human and mouse primary cells 10,000 cells were plated in duplicate for each drug concentration. For cell line CFU assays, MV4-11 cells were treated with drugs in the presence of 20 ng/mL IL-3, GM-CSF, or FGF2. Cells were plated in MethoCult H4230 (Stemcell Technologies) in duplicates at 250 cells/plate. Plates were placed in a 37°C incubator for 7 or 14 days for human cell lines and for mouse or human primary cells, respectively. Colonies were identified as clusters containing 40 or more cells. For CFU assays using human primary CD34^+^ cells, umbilical cord blood was obtained from the OSU Comprehensive Cancer Center (CCC) Leukemia Tissue Bank Shared Resource, and mononuclear cells were isolated by using density gradient centrifugation. CD34^+^ cells were purified using the CD34 UltraPure MicroBead Kit (Miltenyi Biotec) according to the manufacturer’s instructions. CFU assay with CD34^+^ cells was performed as described above, and the total number of colonies were counted after 7–10 days.

### Xenograft models.

TP-0903 was formulated in 5% (w/v) vitamin E TPGS (Antare Health Products) and 1% Tween 80 (Sigma-Aldrich). Gilteritinib was formulated in 0.5% methylcellulose (Sigma-Aldrich). Engraftment and growth of leukemic cells were monitored by noninvasive bioluminescence imaging in cell line xenograft models, as previously done (47). Details on the cell engraftment and dosing in mouse models are provided in the Supplemental Methods.

### Statistics.

All indicated statistical analyses were performed with GraphPad Prism software V7 using either unpaired 2-tailed Student’s *t* test, 1-way ANOVA with Tukey multiple comparison test, 1-way ANOVA with Dunnett’s multiple comparison test to a set control, or log rank test. *P* < 0.05 was determined to be statistically significant. Data represent the mean ± SEM.

### Study approval.

All animal studies were reviewed and approved by the OSU IACUC prior to them be conducted. Deidentified primary patient samples were obtained through the OSU CCC Leukemia Tissue Bank Shared Resource, under their IRB-approved protocol and honest broker status; written informed consent was obtained from all patients prior to use of samples.

## Author contributions

JYJ, DRB, DAG, MN, EDE, KMH, RHW, SJO, BC, ES, and LTB performed the cellular and molecular biology experiments. JYJ performed the in vivo studies, and DRB, DAG, EDE, and MEZT helped during the studies. CJW, SLW, RL, JSB, EH, JCB, BB, and SDB provided reagents and/or expertise. JYJ, DRB, and SDB wrote the manuscript. All authors reviewed and edited the manuscript.

## Supplementary Material

Supplemental data

Supplemental Table 2

Supplemental Table 4

## Version 1 12/03/2020

Electronic publication
