# Supplementary material for: TP-0903 is active in models of drug-resistant acute myeloid leukemia
Source: JCI Insight. 2020 Dec 3;5(23):e140169. doi: 10.1172/jci.insight.140169 (PMC7714403; doi:10.1172/jci.insight.140169)
Supplement: Supplemental data [file jciinsight-5-140169-s137.pdf]

Supplementary materials to:

TP-0903 is active against models of drug-resistant acute myeloid leukemia

Jae Yoon Jeon[1], Daelynn R. Buelow[1], Dominique A. Garrison[1], Mingshan Niu[1], Eric D. Eisenmann[1], Kevin M. Huang[1], Megan E. Zavorka Thomas[1], Robert H. Weber[1], Clifford J. Whatcott[2], Steve L. Warner[2], Shelley J. Orwick[3], Bridget Carmichael[3], Emily Stahl[3], Lindsey T. Brinton[3], Rosa Lapalombella[3,4], James S. Blachly[3,4], Erin Hertlein[3,4], John C. Byrd[1,3,4], Bhavana Bhatnagar[3,4], Sharyn D. Baker[1,3,4]

[1]Division of Pharmaceutics and Pharmacology, College of Pharmacy, The Ohio State University, Columbus OH; [2]Sumitomo Dainippon Pharma Oncology; [3]Division of Hematology, Department of Internal Medicine, The Ohio State University, Columbus OH; [4]Comprehensive Cancer Center, The Ohio State University, Columbus OH

Methods

Cell viability of primary samples

HS5-GFP human bone marrow stromal cells were plated and cultured for 72 hours to near confluence. MOLM13 cells were then seeded onto HS5-GFP cells to establish a co-culture. Twenty-four hours later, cells were treated with increasing concentrations of drug for an additional 72 hours. MOLM13 and HS5-GFP cells were then separated and MTT assays were performed for cell viability assessment of each cell population.

For murine or human primary AML cells, 100,000 cells/well were plated in a 96-well plate and treated with increasing concentrations of drug. Cell viability, via trypan blue, was measured every 24 hours. If the viability fell below 50% or at 72 hours of treatment, whichever came earlier, cell viability of each plate was measured via CellTiter-Glo. Primary human AML cells were obtained from the OSUCCC Leukemia Tissue Bank Shared Resource.

For primary human fibroblasts, cells were plated at 10,000 cells/well in a 96-well plate and cultured for 24 hours. After 24 hours, cells were treated with drug for 72 hours. A MTT assay was performed to assess cell viability.

Targeted gene sequencing

Primary AML samples were analyzed by targeted gene sequencing of 80 genes, as previously described (1). Samples were pooled and analyzed on a MiSeq system using the Illuminia MiSeq Reagent Kit v3. Illumina Isis Banded Smith Waterman aligner and hg19 genome were used to align the sequences. Single nucleotide variant (SNV) and indel calling were performed using MuTect and Varscan2. A variant allele fraction (VAF) cut-off of 0.10 was set for reporting mutations. SNV that are reported as pathogenic SNP were considered mutations. All other SNV needed to be absent from 1000 Genome database, dsSNP137 or dsSNP142. Visual inspection of all variants was carried out using Integrative Genomics Viewer (Broad Institute).

In vivo xenograft mouse models

Male or female 8-12 week-old NSG mice were procured from either Jackson Laboratories (stock number: 005557; Bar Harbor, ME) or Ohio State University (OSU) Comprehensive Cancer Center Target Validation Shared Resources. For cell line xenograft model, animals were intravenously injected with 1 million MOLM13-

Luc+ or MOLM13-Res-Luc+ cells, 0.5 million OCI-AML3-Luc+ cells, and 10,000 Ba/F3-FLT3-ITD/F691L-GFP+ cells. Weekly tumor engraftment of cell line xenograft models were assessed after injection of D-luciferin (150mg/kg i.p.; Gold Biotechnology, Inc, St. Louis, MO) on Xenogen IVIS-200 imaging system (Perkin Elmer, Waltham, MA) or GFP monitoring by peripheral blood via BD LSR II flow cytometer (BD Biosciences, San Jose, CA). Signal intensity was analyzed using Living Image Software (PerkinElmer) or FlowJo v10.0.08 software (FlowJo, LLC, Ashland, OR). Mice were randomized to treatment groups based on signal intensity and leukemia burden. Drug treatments were started on days 3, 7, or 13 after cell injection in OCI-AML3, MOLM13, and MOLM13-Res xenografts, respectively. In murine AML transplantation model, 1 million FLT3-ITD+/-/IDH2-R140Q+/- splenocytes (in house colony) were intravenously injected in male NSG mice. On day 7, mice were started on drug treatments. All mice were observed daily and humanely euthanized when showing signs of progressive disease including, hind limb paralysis, weight loss more than 20%, and lethargy. All animal studies were approved by the OSU Institutional Animal Care and Use Committee.

Apoptosis and CD11b expression assays

Cells were treated with DMSO or 20 nM TP-0903 for up to 48h. At indicated time points, cells were collected and washed with PBS. CD11b expression was measured using anti-CD11b-APC antibody (BioLegend, San Diego, CA). Each sample was suspended in 100uL of FACS buffer (PBS + 4% FBS) with 1uL of antibody, incubated on ice for 15 minutes in the dark and washed once in PBS. DAPI (final concentration 1ug/mL [ThermoFisher Scientific]) was added to each sample as a viability dye. For apoptosis assay, cells were incubated with annexin V-APC (BioLegend) according to manufacturer's instructions. CD11b expression and apoptosis were measured by BD LSR II flow cytometer. Two-color cytometry was performed, and data were analyzed using FlowJo.

Cell cycle analysis

Asynchronous cells were treated with DMSO or 20 or 30 nM TP-0903 for up to 72 h. At indicated time points, cells were collected and washed with 0.1% EDTA PBS (Ricca Chemical Company, Arlington, TX) followed by only PBS. Cells were then fixed on ice with ice-cold 70% ethanol for 30 minutes. Cells were either stored at -20°C for up to 1 month or processed immediately. Cells were spun down at 450 x g for 10 minutes and stained

with DAPI in 0.1% Triton-X (ThermoFisher Scientific) PBS for 30 minutes at room temperature and protected from light. The DNA content was determined using a BD LSR II flow cytometer. The cell cycle distribution was analyzed using FlowJo.

Cytospin slides

Cells that grow out of the CFU assays were washed with PBS twice to remove MethoCult. 100,000 cells were spun down on the slides using cytology funnels (Fisherbrand) in a cytocentrifuge at 600 rpm for 5 minutes. Slides were then air dried overnight and stained with Hema 3 Stain Set (Thermo Fisher) according to manufacturer's instructions. Images of the slides were taken at the Campus Microscopy & Imaging Facility Shared Resources here at OSUCCC using the light microscopy Zeiss Axioskop system.

Inducible MOLM13 NRAS G12D Cells

NRAS cDNA was cloned into pLVX-TetOne-Puro vector (Takara Bio USA). Site-directed mutagenesis to generate G12D mutation was carried out using the QuikChange II XL Site-Directed Mutagenesis Kit (Aligent). Mutagenesis primers were designed using the QuikChange Primer Design program and synthesized by Integrated DNA Technologies (IDT). Lentiviral production was carried out using the Lenti-X Packaging Single Shots (Takara Bio USA) according to manufactures protocol. Viral supernatant was then concentrated using PEG-*it* virus precipitation solution (System Biosciences). MOLM13 AML cells were transduced using a spinfection. Briefly, concentrated viral supernatant was preloaded onto retronectin-coated 6-well plates, 2 × 10^6 cells per well were then added and incubated 72 hours. Media was supplemented with Tet-Free FBS, 10 µg/ml DEAE dextran, and antibiotics. Cells were placed under 2ug/mL puromycin selection for 7 days. Cells were then removed from puromycin and allowed to grow up. Cells were maintained in Tet-Free FBS supplemented media. *NRAS* expression was induced in stably transduced MOLM13 *NRAS* G12D cells with 0.1ug/mL of doxycycline for 24 hours and then maintained at the same concentration for the duration of the experiment. To establish growth curves, cells were seeded in triplicate at a concentration of $1.5x10^5$ cells/mL in 3mL at treated with DMSO, or 15 nM of TP-0903 or gilteritinib. Cells were counted every 2-3 days via Trypan Blue and normalized to Day 0 counts.

RNA isolation and RT-PCR

RNA was isolated from cells using Trizol-Cholorform extraction. cDNA was generated from 0.5ug of RNA using the SuperScript IV First-Strand Synthesis System (ThermoFisher). 50ng of cDNA was used to carry out real time PCR for on target genes, as well as the housekeeping target, GAPDH using TaqMan FAST methodology (ThermoFisher). Target C_t (threshold cycles) was standardized to the GAPDH C_t and graphed as $2^{-\Delta Ct}$.

Supplementary Table 1. Binding affinities (Kd) of TP-0903 and other FLT3 TKIs against FLT3

	Kd (nM)						
	TP-0903	Gilteritinib	Midostaurin	Sorafenib	Quizartinib	Crenolanib	Ponatinib
WT	0.93	1.9	2.6	13	1.5	0.15	0.19
ITD	5.6	0.91	3.2	95	8.5	0.26	4.5
D835H	1.9	0.86	1.3	11	2.3	0.16	4.7
D835V	2.1	0.15	1.5	140	5.6	3.3	3.5
D835Y	1.4	0.65	1.4	24	11	0.14	3.8
ITD/ D835V	0.79	0.12	2.3	630	340	3.6	1100
ITD/ F691L	1.9	0.32	2.7	860	83	22	6.4

Supplementary Table 3. Summary of IC50 values from viability assays

		IC50 (nM)						
Cells/samples	Mutations or added supplements (VAF)	TP-0903	Gilteritinib	Midostaurin	Sorafenib	Quizartinib	Crenolanib	Ponatinib
**Cell Lines**								
MOLM13	ITD	21	17	39	26	2.5	7.4	
MOLM13-Res	ITD/D835Y	16	11	41	1790	400	11	
MV4-11	ITD	17	8	51	8	1.7	6.6	
	GFP+IL3	47	972	328	3848	>10,000	773	920
	ITD	15	5.4	4.2	1.4	0.15	1.8	2.8
	D835H	16	0.5	1.2	76	2.2	0.27	89
Ba/F3	D835Y	22	22	1.5	233	11.7	0.068	158
	ITD/D835H	16	20	28	463	28	38	126
	ITD/D835Y	20	55	33	3337	199	35	106
	ITD/F691L	16	193	20	1339	232	58	41.9
**Primary Samples**								
**FLT3-ITD**								
Murine	IDH2-R140Q	300	2600	10,700	>10,000	>10,000	3100	
Murine	MLL-PTD	400	1550					
142	NPM1 (0.41)	35	334	250				
370	IDH1 (0.7), NPM1 (0.44), SMARCA2 (0.44)	26.6	239	249	1031	375	192	
774	CBL (0.47), IDH1 (0.44), NPM1 (0.42),	45	161	487			659	
240	IDH2 (0.52), NPM1 (0.43)	26.2	92.5	213	1485	238	90	
1355	NPM1 (0.5), IDH2 (0.46)	26.8	105	222	3534	400	305	
2656	IDH2 (0.44), NPM1 (0.43)	33	633	470				
1069	SRSF2 (0.51), IDH2 (0.47), DNMT3A (0.43),	67	286	794	357	195	488	
0762	NRAS (0.16), KRAS (0.03), DNMT3A (0.5), NPM1 (0.44), D835E (0.04)	38	1307	1220	195	357	547	
**FLT3 wild-type**								
003	NRAS (0.27), EZH2 (0.63), JAK3 (0.56), IDH2 (0.42), BCOR (0.4), DNMT3A (0.31)	58	576	1880				
036	NRAS (0.44), IDH2 (0.47), NPM1 (0.47), DNMT3A (0.44),	21	731	334				
398	NRAS (0.45), CCND2 (0.47), ATM (0.4), IDH2 (0.37), STAG2 (0.18)	60	260	490				
**MOLM13 co-culture**								
MOLM13		31	17	39	14	1.5	5	
MOLM13 + HS5-GFP		53	84	99	70	4.7	20	
**MOLM13 supplemented with cytokines**								
MOLM13		7.4	7.9	18.9				
MOLM13	IL3	6.9	11.9	34.4				
MOLM13	IL6	9.4	10.7	58.5				
MOLM13	GM-CSF	10.1	14.8	47.4				
MOLM13	IL3, IL6, GM-CSF	8.1	18.4	54.8				

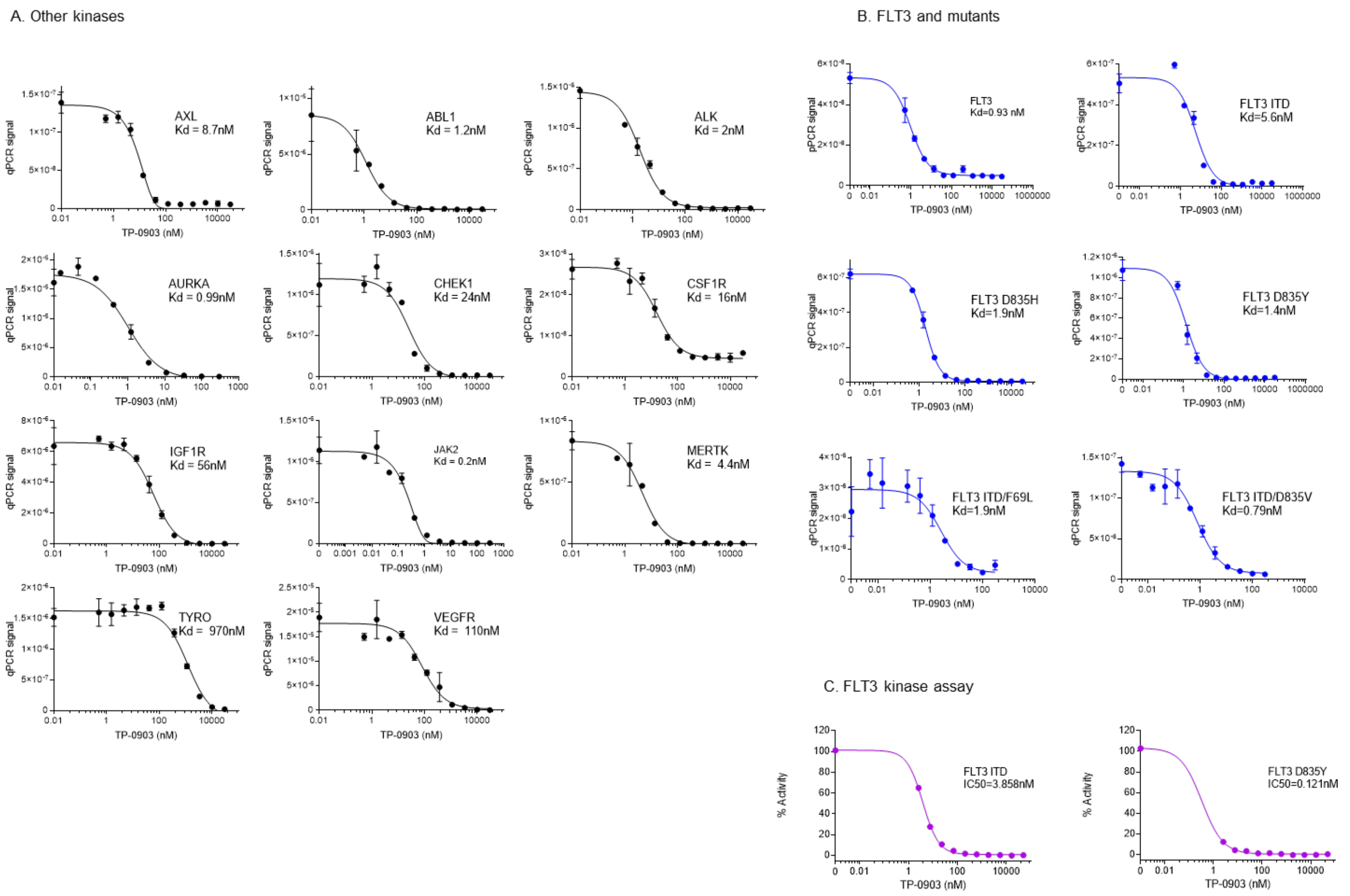

Supplementary Figure 1. Biochemical activity of TP-0903 against different kinases. Activity of TP-0903 against: (**A**) different kinases, (**B**) FLT3 mutants in a binding assay by KdELECT, (**C**) FLT3-ITD and D835Y in an enzymatic kinase assay by Reaction Biology. The corresponding Kd and IC50 shown in nM, respectively.

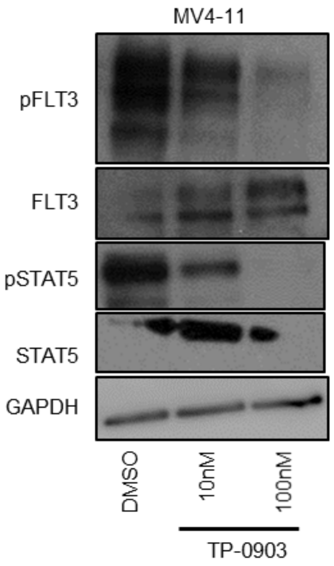

Supplementary Figure 2. Inhibition of FLT3 signaling by TP-0903. Signaling inhibition in MV4-11 cells treated with DMSO or increasing concentrations of TP-0903 for 4 hours. Western blot analysis was performed on whole-cell lysates with the indicated antibodies and is a representative of two independent experiments.

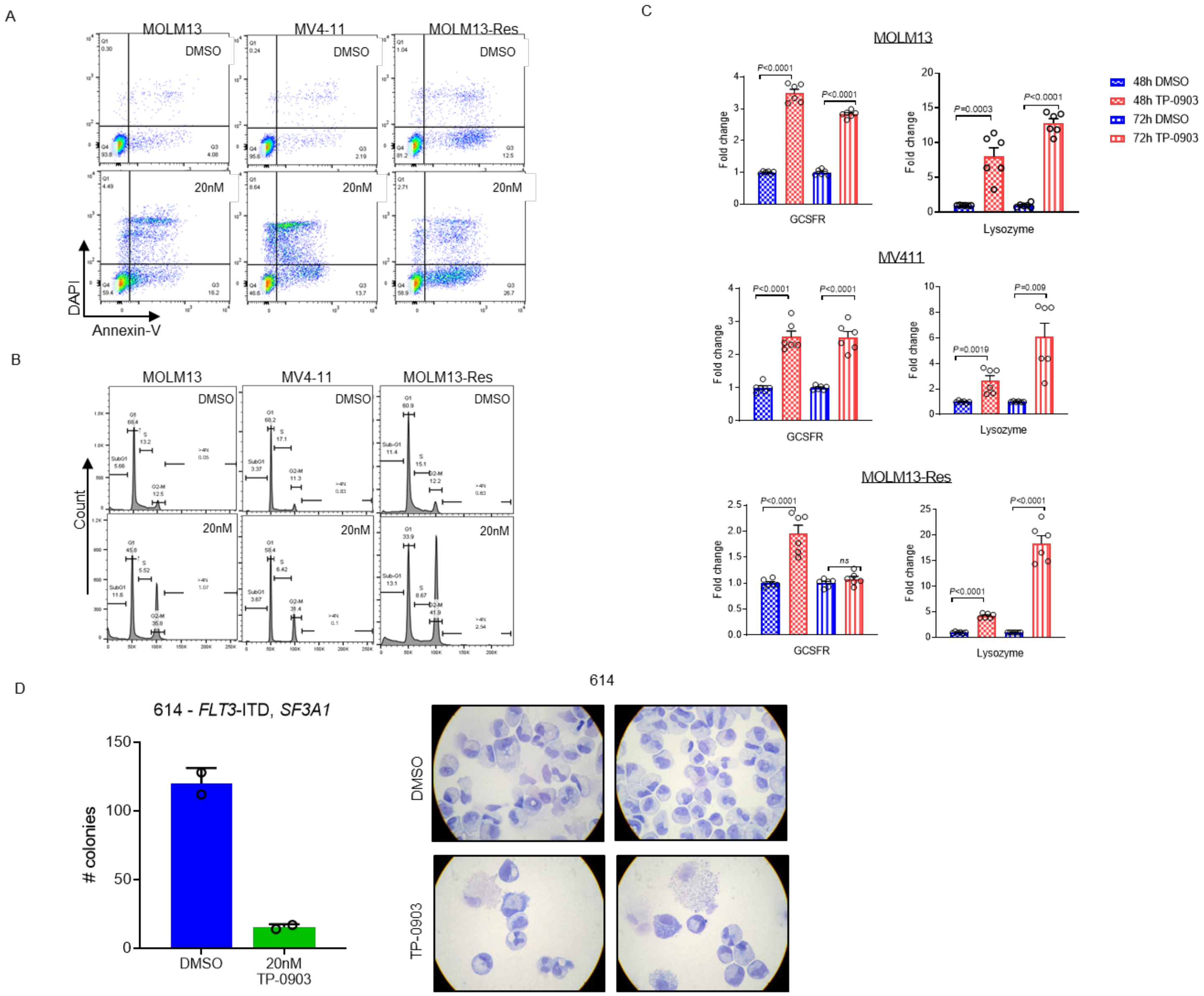

Supplementary Figure 3. Induction of apoptosis, cell cycle arrest and differentiation by TP-0903. (**A**) Representative flow cytometry measurement of apoptosis using annexin-V and DAPI staining at 48h of treatment. (**B**) Representative DAPI histograms of cell cycle phase after 12h of treatment via flow cytometry. (**C**) RT-PCR showing increase in GCSFR and lysozyme gene expression after 48h and 72h of TP-0903 treatment (n=6). (**D**) Inhibition of colony formation (left) and morphology (right) of *FLT3*-ITD+ primary AML sample with indicated mutations treated with TP-0903 (14 day CFU and Wright Giemsa staining, N =2).

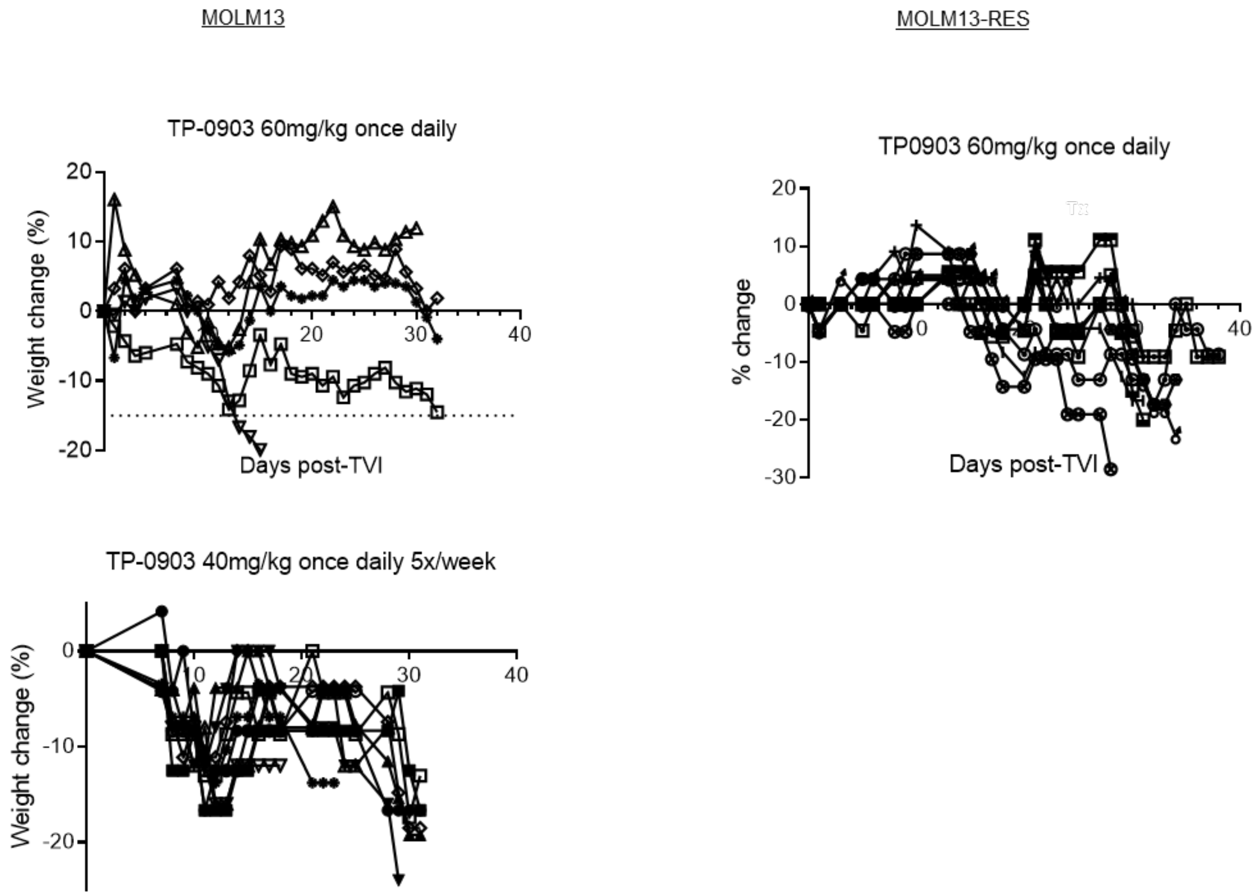

Supplementary Figure 4. TP-0903 tolerability studies. TP-0903 60mg/kg once daily or 40mg/kg 5 days/week, along with vehicle, was administered in female NSG mice engrafted with MOLM13-Luc+ and MOLM13-Res-Luc+ cells. Treatment started on day-7 and -13 post-tail vein injection (TVI) in MOLM13-Luc+ and MOLM13-Res-Luc+, respectively. Change in their daily weights (%) were followed for tolerability.

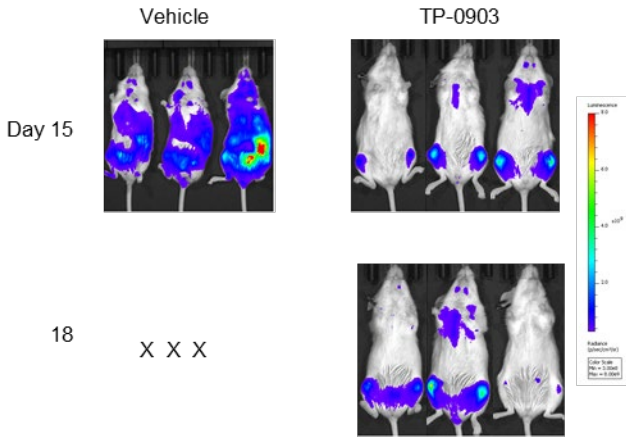

Supplementary Figure 5. Bioluminescence imaging of a MOLM13 cell xenograft in mice. Female NSG mice were engrafted with MOLM13-Luc+ cells and treated with vehicle or TP-0903 60mg/kg once daily for three weeks. Whole body imaging with luciferin was performed on day 15 and 18. Representative images are shown.

B

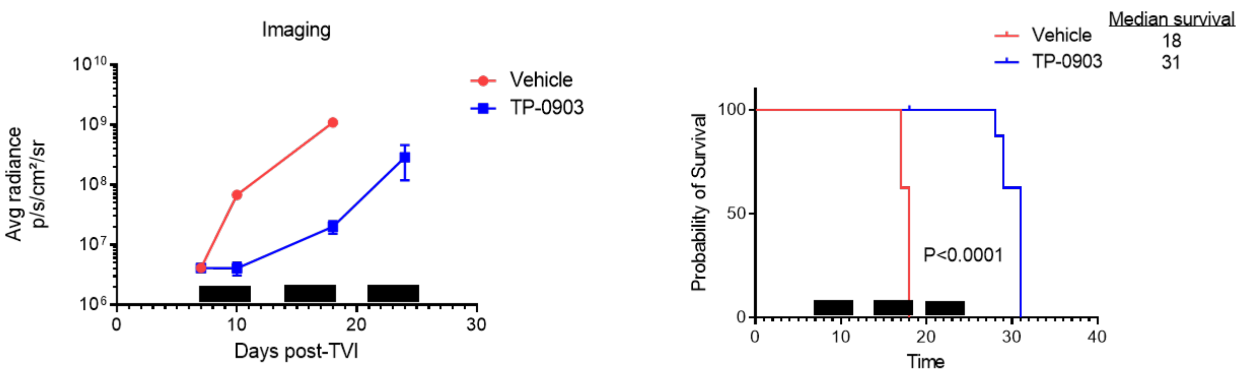

Supplementary Figure 6. TP-0903 is active *in vivo* in a MOLM13 cell xenograft in mice. Bioluminescence signal (mean ± SEM) (top panel) and survival (Kaplan-Meier analysis) (bottom panel) following treatment with TP-0903 60mg/kg 5 days/week or vehicle (N=8-10/cohort). Black bars depict treatment days.

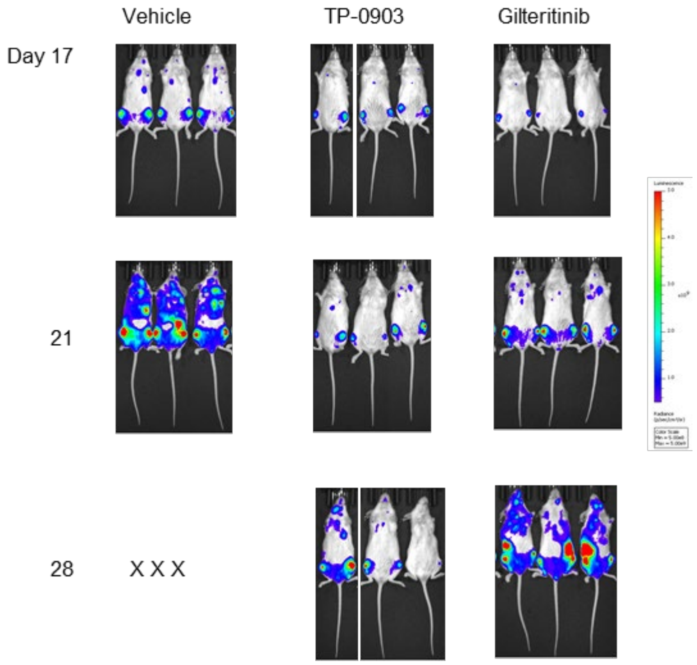

Supplementary Figure 7. Bioluminescence imaging of a MOLM13-RES cell xenograft in mice. Female NSG mice were engrafted with MOLM13-Res-Luc+ and treated with vehicle, TP-0903 60mg/kg, or gilteritinib 30mg/kg 5days/week for three weeks. Whole body imaging was performed on day 17, 21 and 28. Representative images are shown.

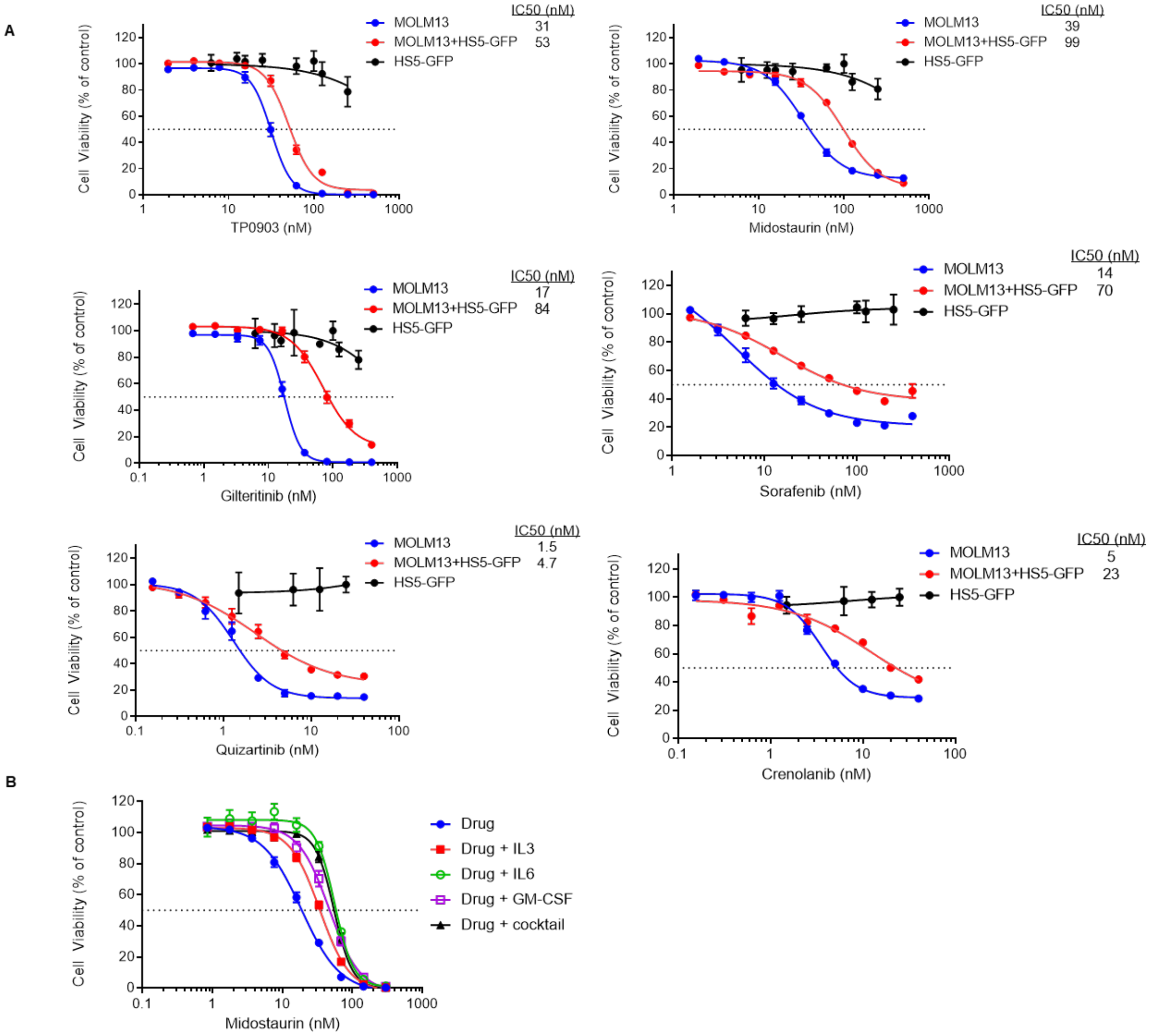

Supplementary Figure 8. Co-culture of MOLM13 cells with HS5-GFP human bone marrow mesenchymal stromal cells (MSCs) and cytokines. Inhibition of MOLM13 cell viability ± (**A**) co-culture with HS5-GFP MSCs with treated with different TKI (MTT assay, 72 h, N=18-24) (**B**) cytokines treated with midostaurin (MTT assay, 72h, N=18-24).

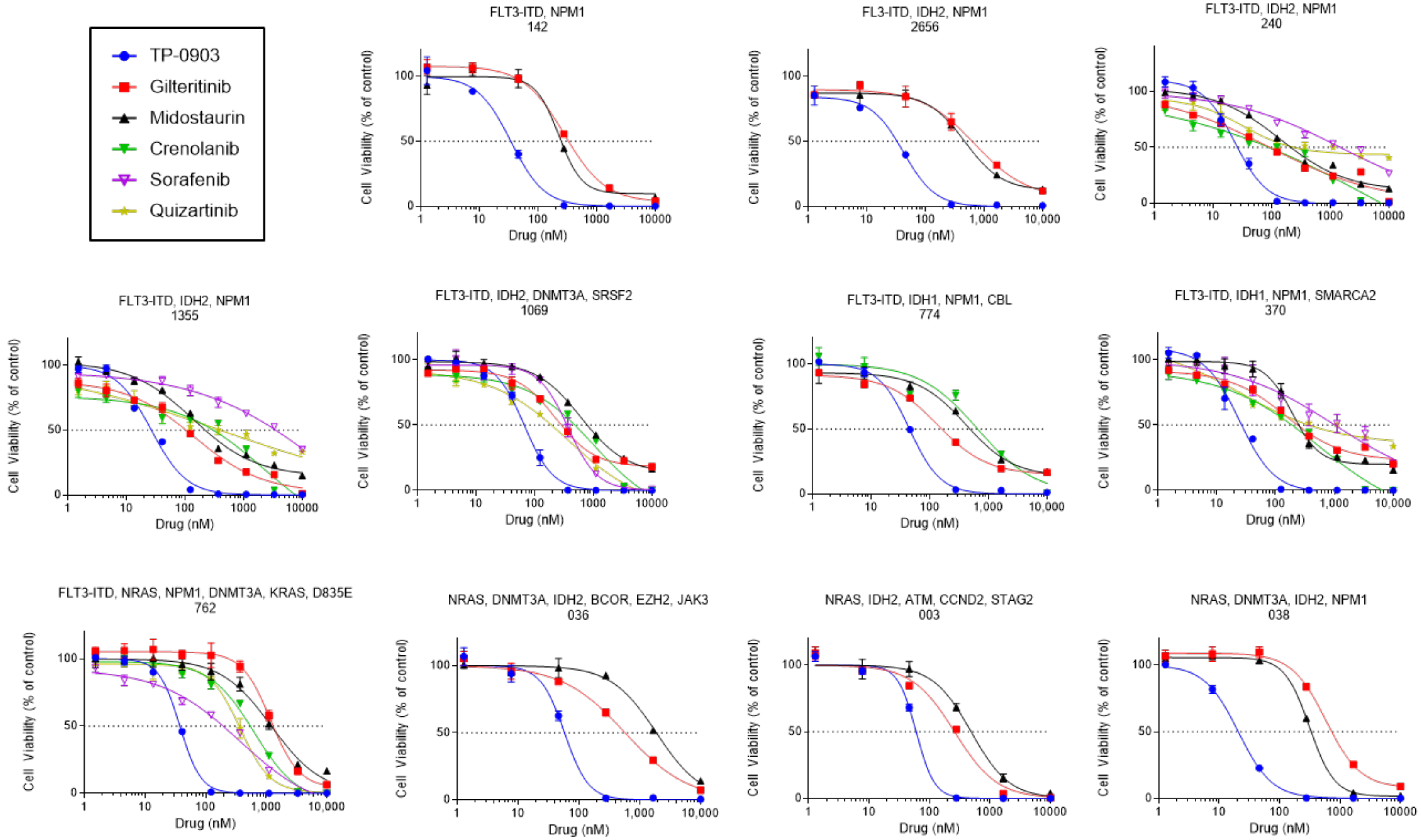

Supplementary Figure 9. TKI activity in human primary AML samples. Inhibition of human primary AML sample's cell viability by panel of TKIs (right corner) (CellTiter Glo assay, 72h, N=3-6). Co-occurring mutations are indicated above each cell viability curve.

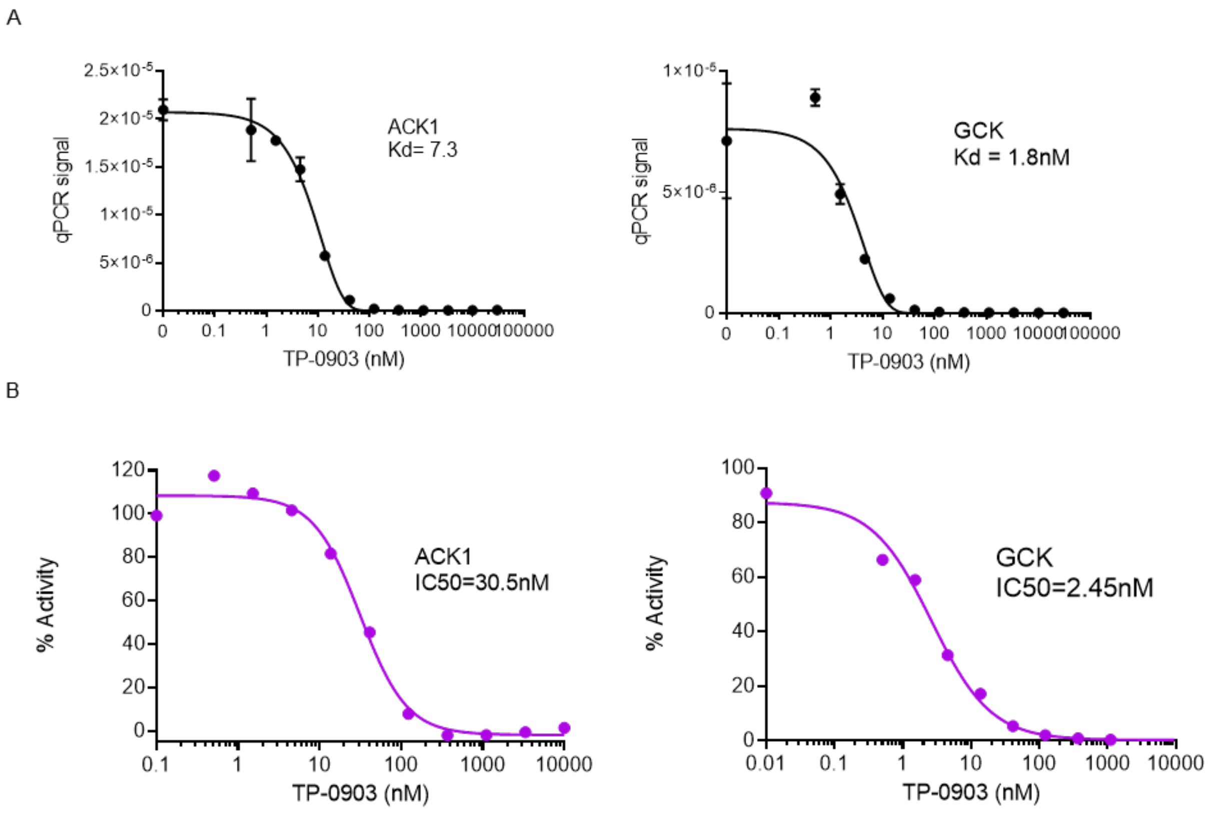

Supplementary Figure 10. Biochemical activity of TP-0903 against ACK1 and GCK. Activity of TP-0903 against ACK1 and GCK in a (**A**) binding assay by KdELECT and (**B**) kinase assay by Reaction Biology. The corresponding Kd and IC50 shown in nM, respectively.

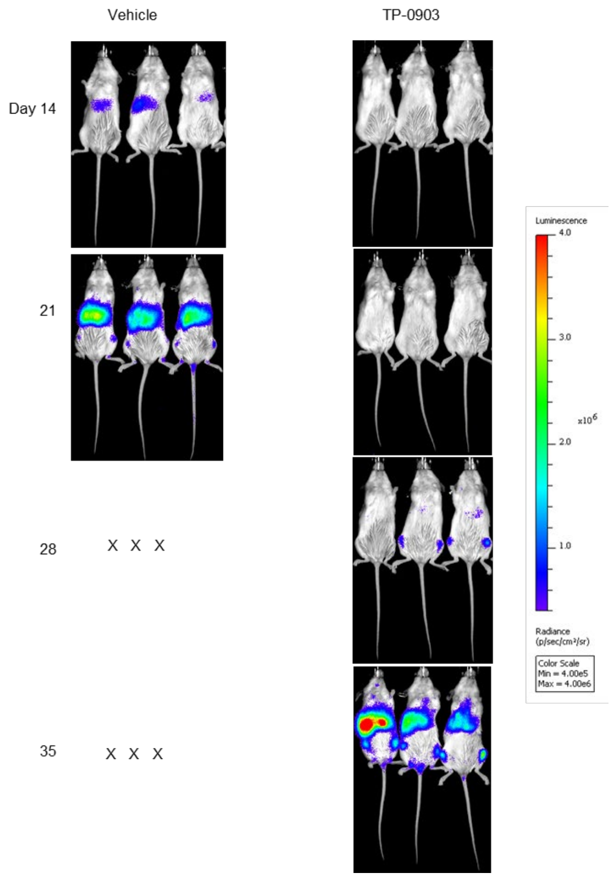

Supplementary Figure 11. Bioluminescence imaging of OCI-AML3 cell line xenograft in mice. Female NSG mice were engrafted with OCI-AML3-Luc+ and treated with vehicle or TP-0903 50mg/kg 5 days/week for 6 weeks. Whole body imaging was performed on day 14, 21, 28, and 35. Representative images are shown.

A

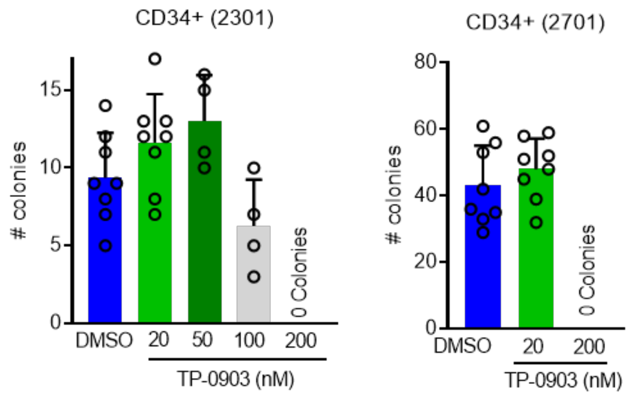

B

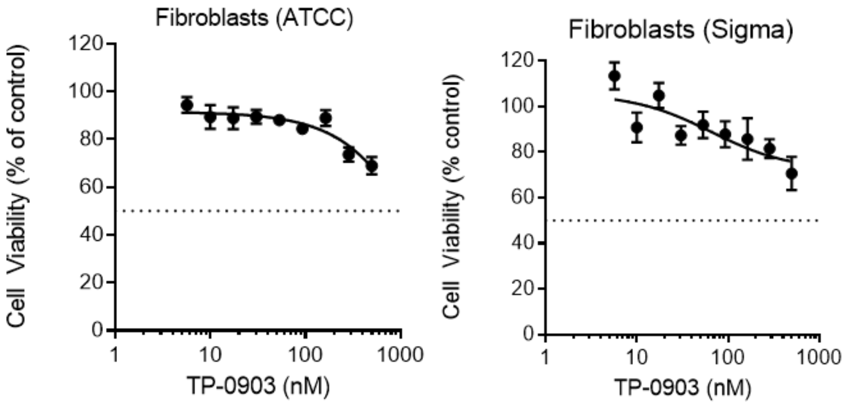

Supplementary Figure 12. **Effect of TP-0903 on normal human cells**. (**A**) Human cord blood CD34+ cells from two different donors were treated with TP-0903 or DMSO and toxicity was assessed in a CFU assay (14 day treatment, N=4-8). (**B**) Human dermal fibroblasts from 2 different donors were treated with TP-0903 or DMS0 and effect on cell viability was determined by MTT assay (72 h treatment, N=6-12).

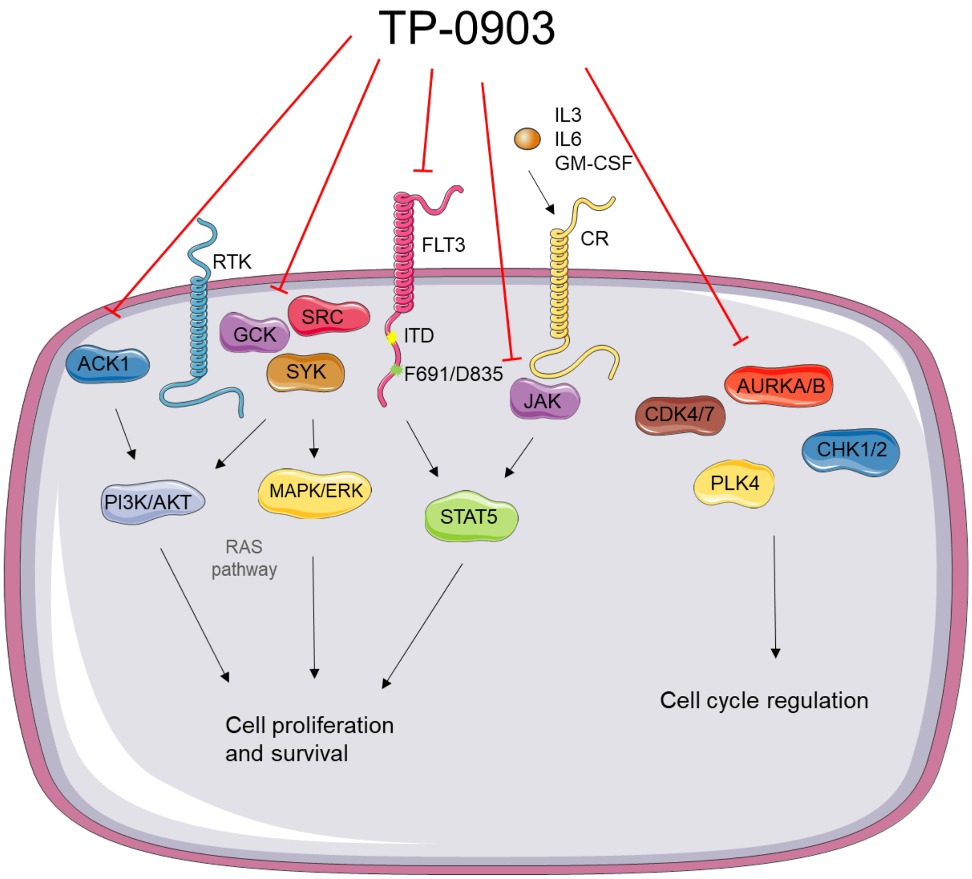

Supplementary Figure 13. Model of TP-0903 activity against multiple mechanisms of drug resistance. Presumed targets inhibited by TP-0903 leading to inhibition of cell proliferation, survival and disruption in cell cycle regulation. Pathway to terminal differentiation of AML cells is not depicted here.

References

1. Eisfeld AK, Kohlschmidt J, Mrozek K, et al. Adult acute myeloid leukemia with trisomy 11 as the sole abnormality is characterized by the presence of five distinct gene mutations: MLL-PTD, DNMT3A, U2AF1, FLT3-ITD and IDH2. *Leukemia*. Nov 2016;30(11):2254-2258.
2. Doench JG, Fusi N, Sullender M, et al. Optimized sgRNA design to maximize activity and minimize off-target effects of CRISPR-Cas9. *Nat Biotechnol*. Feb 2016;34(2):184-191.
3. Joung J, Konermann S, Gootenberg JS, et al. Genome-scale CRISPR-Cas9 knockout and transcriptional activation screening. *Nat Protoc*. Apr 2017;12(4):828-863.
4. Jiang H, Lei R, Ding SW, Zhu S. Skewer: a fast and accurate adapter trimmer for next-generation sequencing paired-end reads. *BMC Bioinformatics*. Jun 12 2014;15:182.
5. Li W, Xu H, Xiao T, et al. MAGeCK enables robust identification of essential genes from genome-scale CRISPR/Cas9 knockout screens. *Genome Biol*. 2014;15(12):554.
6. *MAGeCKFlute: Integrative analysis pipeline for pooled CRISPR functional genetic screens.* . Version R package version 1.4.3. 2020.
